# Development of a bispecific nanobody conjugate broadly neutralizes diverse SARS-CoV-2 variants and structural basis for its broad neutralization

**DOI:** 10.1371/journal.ppat.1011804

**Published:** 2023-11-30

**Authors:** Jing Yang, Sheng Lin, Zimin Chen, Fanli Yang, Liyan Guo, Lingling Wang, Yanping Duan, Xindan Zhang, Yushan Dai, Keqing Yin, Chongzhang Yu, Xin Yuan, Honglu Sun, Bin He, Yu Cao, Haoyu Ye, Haohao Dong, Xianbo Liu, Bo Chen, Jian Li, Qi Zhao, Guangwen Lu

**Affiliations:** 1 Department of Emergency Medicine, State Key Laboratory of Biotherapy, West China Hospital, Sichuan University, Chengdu, Sichuan, China; 2 Disaster Medicine Center, West China Hospital, Sichuan University, Chengdu, Sichuan, China; 3 Department of Biotherapy, State Key Laboratory of Biotherapy and Cancer Center, West China Hospital, Sichuan University, Chengdu, Sichuan, China; 4 State Key Laboratory of Biotherapy and Cancer Center, National Clinical Research Center for Geriatrics, West China Hospital, Sichuan University, Chengdu, Sichuan, China; 5 CHENGDU NB BIOLAB CO., LTD, Chengdu, Sichuan, China; 6 School of Basic Medical Sciences, Chengdu University, Chengdu, Sichuan, China; 7 College of Food and Biological Engineering, Chengdu University, Chengdu, Sichuan, China; CharitΘ û University Medical School Berlin, Campus Benjamin Franklin, GERMANY

## Abstract

The continuous emergence of severe acute respiratory syndrome coronavirus 2 (SARS-CoV-2) variants with increased transmissibility and profound immune-escape capacity makes it an urgent need to develop broad-spectrum therapeutics. Nanobodies have recently attracted extensive attentions due to their excellent biochemical and binding properties. Here, we report two high-affinity nanobodies (Nb-015 and Nb-021) that target non-overlapping epitopes in SARS-CoV-2 S-RBD. Both nanobodies could efficiently neutralize diverse viruses of SARS-CoV-2. The neutralizing mechanisms for the two nanobodies are further delineated by high-resolution nanobody/S-RBD complex structures. In addition, an Fc-based tetravalent nanobody format is constructed by combining Nb-015 and Nb-021. The resultant nanobody conjugate, designated as Nb-X2-Fc, exhibits significantly enhanced breadth and potency against all-tested SARS-CoV-2 variants, including Omicron sub-lineages. These data demonstrate that Nb-X2-Fc could serve as an effective drug candidate for the treatment of SARS-CoV-2 infection, deserving further *in-vivo* evaluations in the future.

## Author summary

The pandemic of SARS-CoV-2 has led to a severe public health crisis. In view of the wide clinical-applications of antibody drugs, it has always been a research focus to identify new antibodies and/or nanobodies with anti-viral neutralizing activity. In this study, we identified two neutralizing nanobodies (Nb-015 and Nb-021) that target non-overlapping epitopes in SARS-CoV-2 S-RBD with high affinity. Both nanobodies could broadly neutralize diverse SARS-CoV-2 variants. Furthermore, we revealed two distinct neutralization mechanisms of Nb-015 and Nb-021 by high-resolution nanobody/S-RBD complex structures. Eventually, we engineered a bispecific nanobody conjugate which exhibits excellent inhibitory activity against SARS-CoV-2, including the original strain and the VOI and VOC variants. We believe that the nanobody conjugate identified in this study could serve as an effective anti-viral drug reserve for potential treatment of COVID-19.

## Introduction

Severe acute respiratory syndrome coronavirus 2 (SARS-CoV-2), the etiological agent of coronavirus disease 2019 (COVID-19), has led to a global pandemic lasting for over three years. To date, over 757 million people have suffered from the viral infection, leading to more than 6.8 million deaths worldwide (https://covid19.who.int/). SARS-CoV-2 initially binds to the host receptor, angiotensin-converting enzyme 2 (ACE2), by the receptor-binding domain (RBD) of its spike (S) glycoprotein to tether the viral particles to the cell surface [1,2]. This receptor binding process is believed to subsequently trigger the S-mediated membrane fusion, eventually leading to viral entry to set up infection [3,4]. Therefore, the RBD of S (S-RBD) is a major target of the host humoral immunity [5]. Multiple antibodies that target SARS-CoV-2 S-RBD have thus far been developed to fight against the viral infection, and several of them have been approved for clinical treatment of COVID-19 [6–8]. Nevertheless, the emergence of SARS-CoV-2 variants, especially those defined by the World Health Organization as variants of interest (VOIs) or concern (VOCs), has raised new challenges because of their increased transmissibility and immune-escape capacity. Several mutations identified in the VOI and VOC strains are shown to severely compromise the neutralizing efficacy of available therapeutic antibodies [9,10]. Of special note is the Omicron variant that has accumulated more than 30 mutations in S, among which over 15 are located in S-RBD. Consequently, only a limited number of antibodies targeting the CR3022 site or S309 site retain comparable binding towards Omicron S-RBD, whereas antibodies targeting the RBS-A, -B, -C and -D sites are significantly escaped by the virus [11,12]. Thus, there is an urgent and continuing need to identify antibodies able to counter all current SARS-CoV-2 variants including Omicron.

Camelid-derived VHH antibodies, also known as nanobodies, are natural, single-heavy-chain monovalent antibody fragments [13]. In comparison to conventional antibodies, nanobodies represent the smallest antigen-binding domain, therefore featuring with several physicochemical advantages, such as smaller size (12~15 kDa), easier production in different expression systems, higher thermal stability and solubility, etc [14]. In addition, nanobodies can be easily bioengineered into homo/heterodimers or multimers, such as by fusing to an immunoglobulin-G (IgG) Fc tag or simply by linking two or more nanobodies in a tandem manner, to increase the neutralizing potency and reduce the risk for viral escape [15,16]. These unique properties of nanobodies have led them to be developed as potential therapeutics against viral infection, including SARS-CoV-2 infection [17,18].

Here, we report the identification and characterization of two neutralizing nanobodies isolated from an immunized alpaca, Nb-015 and Nb-021, which target two non-overlapping epitopes on SARS-CoV-2 S-RBD. Structural and functional studies reveal two distinct neutralizing mechanisms for Nb-015 and Nb-021. The former recognizes a conserved epitope in the ridge of the S-RBD external subdomain and inhibits viral entry by competing with ACE2 binding, and the latter binds to the inner face of the S-RBD core subdomain and neutralizes viral infection likely by destabilizing the architecture of S trimer. Eventually, a bispecific format of the two nanobodies, Nb-021-(GGGGS)_4_-Nb-015, was constructed and further modified by fusing with an IgG1 Fc tag. The resultant tetravalent nanobody conjugate exhibits significantly improved inhibitory activity against SARS-CoV-2, including the prototype strain and the VOI and VOC variants. These data qualify the tetravalent bispecific nanobody as a candidate for the development of a pan-variant antiviral therapeutic against SARS-CoV-2.

## Results

### Identification of two neutralizing nanobodies (Nb-015 and Nb-021) targeting different epitopes in S-RBD

**Fig 1:**
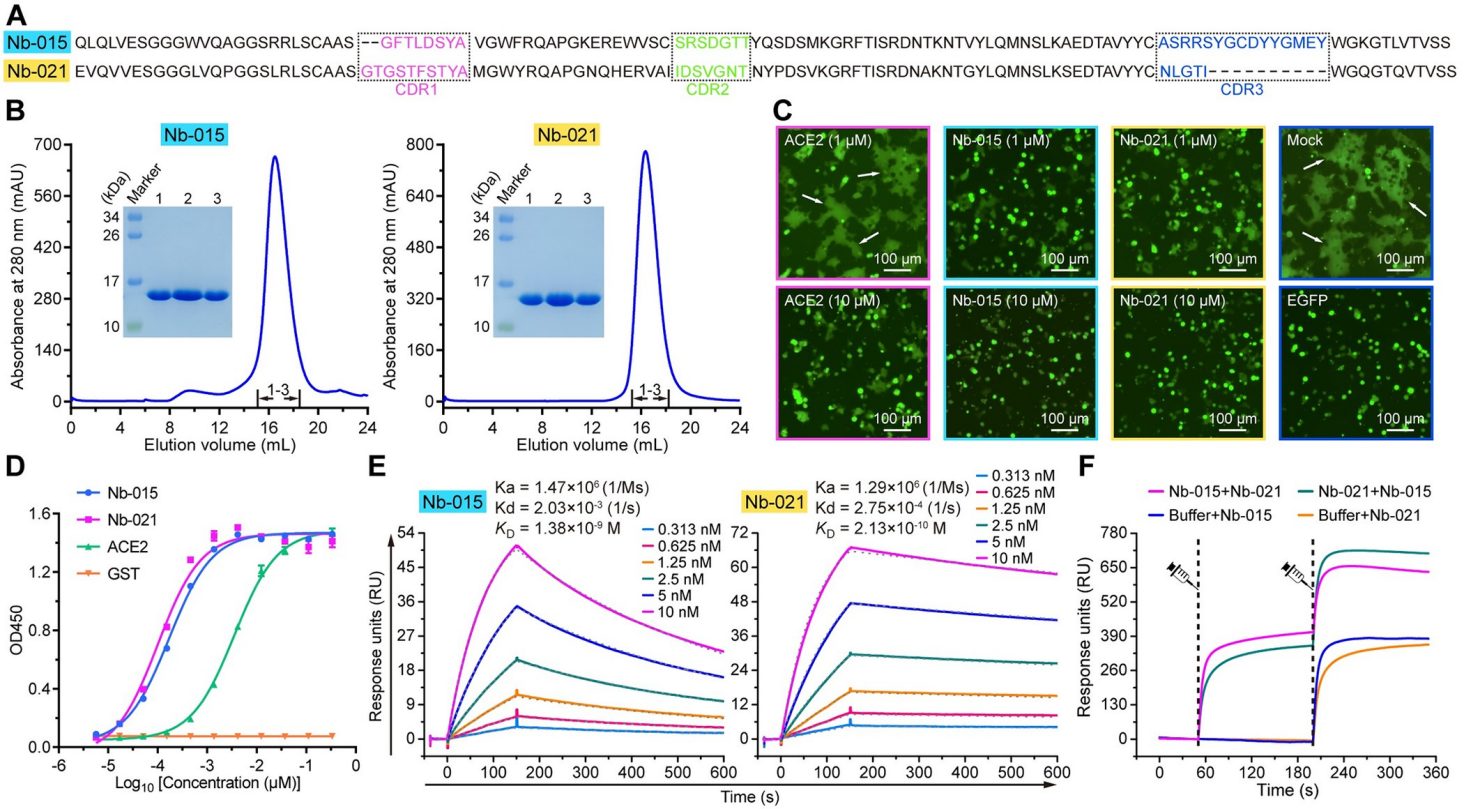
Identification of two neutralizing nanobodies (Nb-015 and Nb-021) targeting two different epitopes on SARS-CoV-2 S-RBD with high affinity. **A,** The amino-acid sequence of Nb-015 and Nb-021. The complementarity-determining region (CDR) 1, CDR2, CDR3 are marked with dashed boxes and colored in magenta, green and blue, respectively. **B,** Solution behavior of Nb-015 and Nb-021 on gel filtration chromatography. The inset figures show the SDS-PAGE analyses of the pooled samples. **C,** Inhibition of SARS-CoV-2 S-mediated cell-cell fusion by Nb-015 or Nb-021 at the indicated concentrations. ACE2 was used as positive control. Mock: syncytium formation induced by mixing HEK293T-ACE2 and HEK293T-S/EGFP cells without the extra addition of any proteins. EGFP: only 293T-S/EGFP cells. The representative syncytia are labelled with white arrows. Scale bar equals 100 μm. **D,** Multi-concentration ELISA-binding assay of the indicated proteins (Nb-015, Nb-021, ACE2 and GST) towards SARS-CoV-2 S-RBD. The OD450 emissions are depicted by curves. Error bar represents the mean ± SD of triplicate. **E,** Binding affinity between Nb-015 (the left panel) or Nb-021 (the right panel) and SARS-CoV-2 S-RBD determined by SPR. The binding profiles and the *K*_D_ values are shown. **F,** SPR kinetics of the competitive binding of Nb-015 and Nb-021 to SARS-CoV-2 S-RBD. For all kinetics, S-RBD was immobilized onto CM5 sensor chip, the indicted proteins (Nb-015 and Nb-021) were successively injected. The real-time binding curves are recorded.

To obtain SARS-CoV-2 S-RBD-specific neutralizing nanobodies, we previously immunized one alpaca using the recombinant prototype S-RBD prepared from insect cells. A screening trial targeting ten nanobodies of unique sequences in our previous work enabled us to identify a neutralizing nanobody with superior S-RBD binding capacity [19]. In this study, another 18 uncharacterized nanobodies (named, following the ten previously characterized nanobodies [19], as Nb-011 to Nb-028) were selected from the unique-sequence nanobody repertoire for further characterization (Figs 1A and S1). These nanobodies were individually purified from *brevibacillus* cells (Figs 1B and S2) and then analyzed via a single-concentration enzyme-linked immunosorbent assay (ELISA). As expected, all the nanobodies tested readily interacted with S-RBD (S3 Fig). We therefore subsequently performed the S-mediated cell-cell fusion assay to evaluate their potential cell-cell fusion inhibition activity. The recombinant ACE2 protein, which could function as a decoy to inhibit the S-associated cell-cell fusion [20,21], was selected as a positive control. For the nanobodies screened, apparent inhibition of the syncytium formation was observed for Nb-015 and Nb-021 at both 10 μM and 1 μM concentrations, featuring an inhibitory capacity superior to ACE2 which only showed clear inhibition at 10 μM (Fig 1C). In contrast, the remaining 16 nanobodies showed no obvious fusion inhibitory activity even at a concentration as high as 10 μM (S4 Fig).

Following the initial screening, the binding kinetics of Nb-015 and Nb-021 to S-RBD were further characterized via the multi-concentration ELISA and the surface plasmon resonance (SPR) assay. In consistent with that observed in our syncytium-formation-inhibition experiments, the two nanobodies readily bound to S-RBD and both exhibited much better binding than the decoy ACE2 protein (Fig 1D). The binding affinities of the two nanobodies (used as the analyte) to S-RBD (immobilized on chip) were then quantitatively determined via SPR. The kinetic binding data revealed an equilibrium dissociation constant (*K*_D_) of 1.38 nM for Nb-015 and of 0.213 nM for Nb-021, respectively (Fig 1E). These values represented approximately 65- and 423-fold higher affinity than that observed for ACE2 (ACE2 similarly used as the analyte and S-RBD immobilized on chip) [19]. In parallel, we also evaluated whether Nb-015 and Nb-021 could simultaneously bind to S-RBD via the competitive SPR experiments. The results revealed that the pre-bound of one nanobody to S-RBD would not interfere with the binding by the other nanobody (Fig 1F), indicating that the two nanobodies targeted non-overlapping epitopes on the S-RBD antigen.

### Neutralization of Nb-015 and Nb-021 against SARS-CoV-2 variants

**Fig 2:**
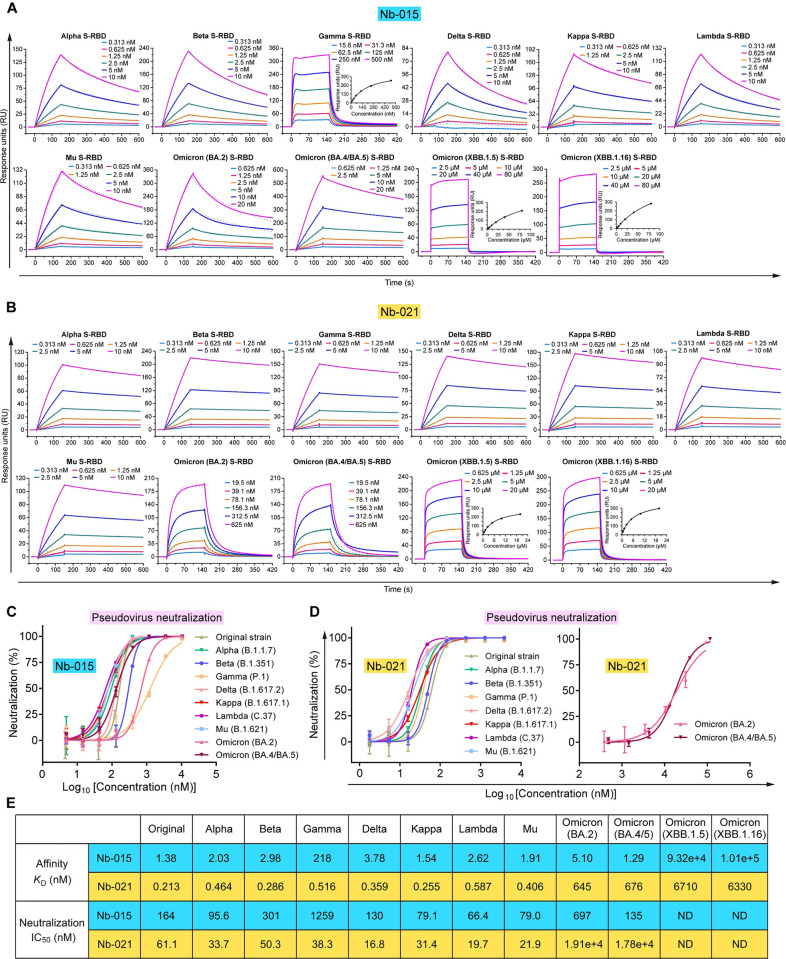
Quantitative analysis of the binding capacity and neutralizing potency of Nb-015 and Nb-021. **A,** SPR assays determine the binding affinity of Nb-015 to the S-RBD from the indicated VOCs and VOIs. S-RBD was immobilized onto a sensor chip. Gradient concentrations of Nb-015 were then flowed over the indicated S-RBD on the chip surface. The kinetic data of Nb-015 binding to Gamma, XBB.1.5 and XBB.1.16 S-RBDs featured with a fast-on/fast-off kinetic and were analyzed using the steady-state affinity model, with the inset figure showing the saturation of the analyte during the SPR test. The other data, with slow-on/slow-off kinetics, were analyzed with the 1:1 binding model. **B,** SPR assays determine the binding affinity of Nb-021 to the S-RBD from the indicated VOCs and VOIs. S-RBD was immobilized onto a sensor chip. Gradient concentrations of Nb-021 were then flowed over the indicated S-RBD on the chip surface. The kinetic data of Nb-021 binding to XBB.1.5 and XBB.1.16 S-RBDs featured with a fast-on/fast-off kinetic and were analyzed using the steady-state affinity model, with the inset figure showing the saturation of the analyte during the SPR test. The other data, with slow-on/slow-off kinetics, were analyzed with the 1:1 binding model. **C,** Neutralizing potency of Nb-015 characterized using SARS-CoV-2 pseudoviruses. Error bar stands for the mean ± SD. Experiments were performed in triplicates. **D,** Neutralizing potency of Nb-021 characterized using SARS-CoV-2 pseudoviruses. Mean ± SD are determined from triplicate experiments and presented with error bars. **E,** A table summarizing the *K*_D_ values and the neutralizing IC_50_ values. “ND” represents “not determined”.

Along with the global spread of SARS-CoV-2, multiple variants have emerged. These still-circulating or once-circulated viral variants, including the known VOC strains (Alpha, Beta, Gamma, Delta and Omicron) and several VOI strains (e.g., Kappa, Lambda and Mu), have led to several waves of re-emerging SARS-CoV-2 pandemics in the past years and therefore have drawn worldwide attention. The residue substitutions identified in S of these variant viruses, especially those accumulated in S-RBD, are shown to cause significant immune escape [22,23]. We therefore further explored the binding capacities of Nb-015 and Nb-021 towards S-RBDs of these variant viruses. The individual S-RBD proteins, including those derived from the Alpha, Beta, Gamma, Delta, Kappa, Lambda, Mu, Omicron (BA.2) and Omicron (BA.4/BA.5), were prepared to high-purity (S5 Fig) and then subjected to SPR analyses for the real-time binding kinetics with the two nanobodies (Fig 2A and 2B). The results revealed that Nb-015 and Nb-021 were able to recognize variant S-RBDs. For Nb-015, it showed strong bindings to Alpha, Beta, Delta, Kappa, Lambda, Mu, Omicron (BA.2) and Omicron (BA.4/BA.5) S-RBDs, with affinities (ranging from 1.29 to 5.10 nM) that are similar or comparable to that when binding to S-RBD of the original strain. Nb-015 also readily interacted with Gamma S-RBD but exhibited a decreased affinity (218 nM) (Fig 2E). For Nb-021, it reserved robust S-RBD binding towards the Alpha, Beta, Gamma, Delta, Kappa, Lambda and Mu strains, with *K*_D_ values ranging from 0.255 to 0.587 nM. Targeting Omicron S-RBDs, however, Nb-021 exhibited dramatically decreased affinities. The *K*_D_ values were determined to be 645 nM towards Omicron (BA.2) S-RBD, 676 nM towards Omicron (BA.4/BA.5) S-RBD, respectively (Fig 2E).

In the next step, we performed the pseudovirus neutralization assay to assess the entry-inhibition efficacy of Nb-015 and Nb-021 against SARS-CoV-2. Echoing our SPR results, both nanobodies could effectively neutralize the viral infection (Fig 2C and 2D). Nb-015 was a potent neutralizer against the prototype, Alpha, Beta, Delta, Kappa, Lambda, Mu, Omicron (BA.2) and Omicron (BA.4/BA.5) viruses. The half maximal inhibitory concentration (IC_50_) values of the nanobody against these viruses were determined to be ranging from 66.4 to 697 nM (Fig 2E). Nb-015 was also able to inhibit viral infection of the Gamma variant but with a moderately compromised activity, showing an IC_50_ of 1259 nM. Regarding Nb-021, potent neutralization was expectedly observed for the original strain and the Alpha, Beta, Gamma, Delta, Kappa, Lambda and Mu variants, with IC_50_ values ranging from 16.8 to 61.1 nM (Fig 2E). Targeting the Omicron sub-lineages, the neutralization efficacy of Nb-021 was dramatically compromised. The IC_50_s of the nanobody against the Omicron (BA.2) and Omicron (BA.4/BA.5) viruses were calculated to be 19.1 μM and 17.8 μM, respectively (Fig 2E). Despite of the decreased neutralization capacity, complete block of the pseudovirus entry by Nb-021 could still be observed at a high nanobody concentration (Fig 2D), indicating that the nanobody remained capable of neutralizing the Omicron (BA.2) and Omicron (BA.4/BA.5) variants.

We also prepared the S-RBD proteins derived from the circulating XBB-lineage viruses (including XBB.1.5 and XBB.1.16) and investigated the binding of our nanobodies towards the XBB proteins (Figs 2A, 2B, and S5). Nb-015 only showed a marginal binding to the XBB S-RBDs, with *K*_D_ values of 93.2 μM for XBB.1.5 S-RBD and 101 μM for XBB.1.16 S-RBD, respectively (Fig 2E). For Nb-021, the binding affinities towards the XBB proteins were determined to be 6.71 μM for XBB.1.5 S-RBD and 6.33 μM for XBB.1.16 S-RBD, respectively (Fig 2E). The XBB mutations therefore would much more severely compromise the spike binding by Nb-015 than by Nb-021.

### Structural basis for the neutralization by Nb-015

**Fig 3:**
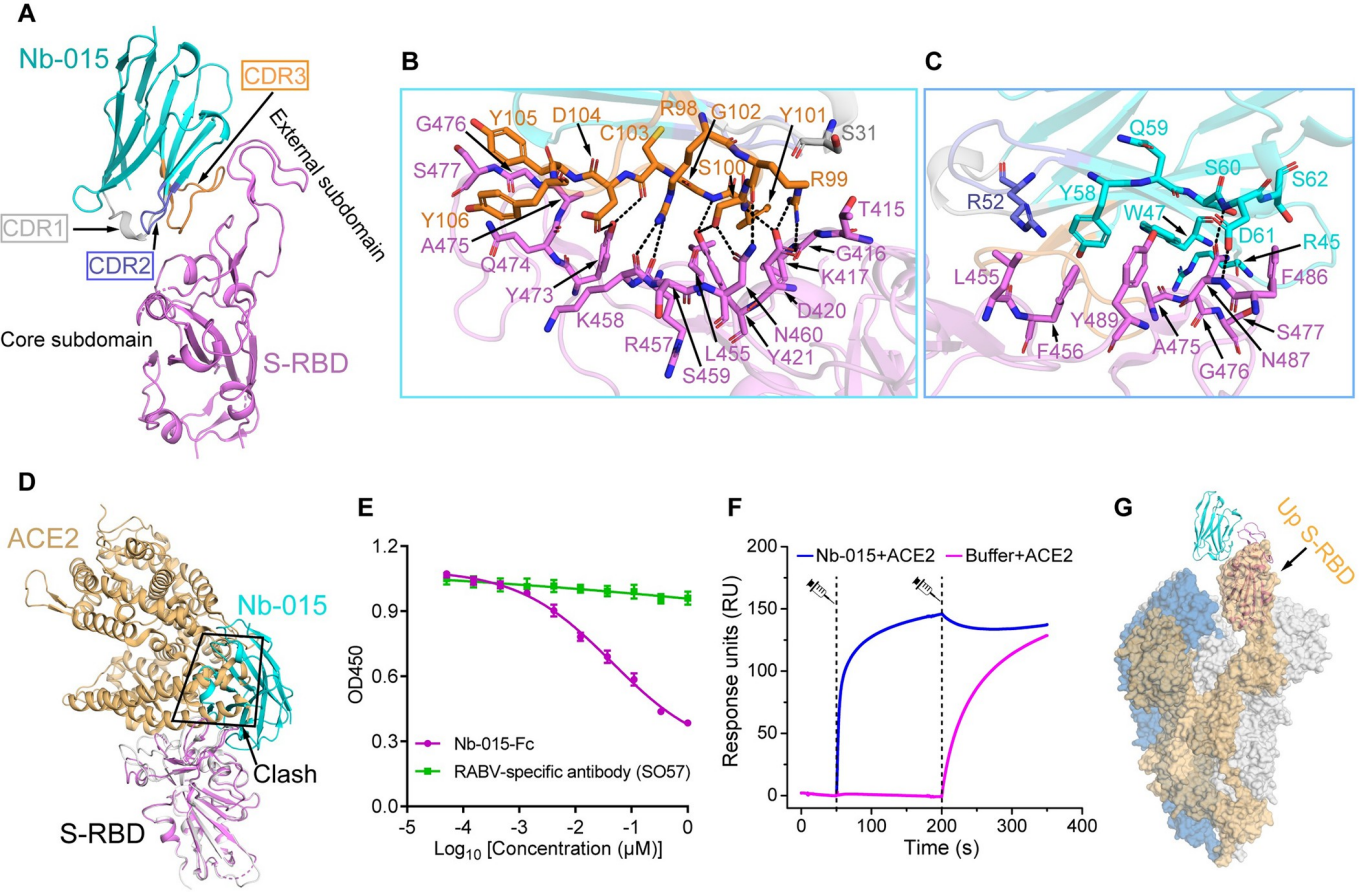
Structural basis for Nb-015 neutralization. **A,** Overall structure of Nb-015/S-RBD complex shown in cartoon. S-RBD is highlighted in magenta and Nb-015 in cyan. The CDR1, CDR2 and CDR3 of Nb-015 are indicated by arrows and colored in gray, lightblue and orange, respectively. The external subdomain and core subdomain of S-RBD are labelled. **B and C,** Detailed interactions between Nb-015 and SARS-CoV-2 S-RBD. **B,** Interactions between the CDR1 and CDR3 of Nb-015 and S-RBD. **C,** Interactions between the CDR2 and framework region of Nb-015 and S-RBD. Residues providing ≥2 van der Waals contacts are shown. The distance cutoff is 4.5 Å. Strong hydrophilic interactions (hydrogen bonds and salt bridges) between Nb-015 and S-RBD (the distance cutoff is 3.1 Å) are presented as dashed lines. **D,** Structural alignment of Nb-015/S-RBD and ACE2/S-RBD complexes [PDB code: 6M0J] [2]. Steric clash between Nb-015 and ACE2 is highlighted. **E,** Competitive binding of Nb-015 and ACE2 to SARS-CoV-2 S-RBD detected by ELISA. S-RBD was coated on 96-well plates, ACE2 was firstly incubated, followed by 3-fold serial dilutions of Nb-015-Fc. Error bars represent the mean ± SD in triplicates. **F,** Competitive SPR assay of Nb-015 and ACE2 binding to SARS-CoV-2 S-RBD. S-RBD was immobilized onto CM5 sensor chip. Nb-015 and ACE2 were flowed over the ship successively. The real-time binding data are shown. **G,** Superimposition of the Nb-015/S-RBD structure (shown in cartoon) onto the SARS-CoV-2 S-trimer structure [PDB code: 6VYB] [72]. The up conformation of the RBD in the S trimer structure (shown in surface) is highlighted.

In order to learn the basis of neutralization by Nb-015, we solved the crystal structure of the nanobody bound to S-RBD at a resolution of 2.0 Å. The structure was determined by molecular replacement method and refined to *R*_work_ = 0.171 and *R*_free_ = 0.202, respectively (S1 Table). In the crystallographic asymmetric unit, it contained a single Nb-015/S-RBD complex bound in a 1:1 binding mode. Overall, Nb-015 mainly utilized its long CDR3 and a small portion of its framework region to interact with a concave epitope located largely at the ridge of the S-RBD external subdomain (Fig 3A). Detailed analyses of the nanobody/S-RBD interactions revealed an extended interface involved Nb-015 residues S31 in CDR1, R52 in CDR2, R98-Y106 in CDR3, R45, W47 and Y58-S62 in the framework region and S-RBD amino acids T415-K417 and D420-Y421 in the core subdomain, and L455-N460, Y473-S477, F486-N487 and Y489 in the external subdomain (Fig 3B and 3C). Among the numerous engagements provided by these residues, a total of thirteen hydrophilic interactions (hydrogen bonds and salt bridges) were observed to form, further stabilizing the nanobody/S-RBD complex (Fig 3B and 3C).

Based on the binding site of Nb-015 on S-RBD, we found that the epitope resembled that targeted by the potent SARS-CoV-2 neutralizing antibody CB6, therefore matching to the RBS-A interface (S6 Fig). Antibodies targeting the RBS-A antigenic site exert neutralization by competing against the S-RBD/ACE2 engagement [24]. Consistently, the footprint of Nb-015 is partially overlapping with the ACE2 binding interface. Of the nineteen epitope residues that are recognized by Nb-015, eight are also involved in ACE2 binding (S7 Fig). Superimposition of the Nb-015/S-RBD and the ACE2/S-RBD structures clearly showed that the nanobody sterically clashed with the bound receptor (Fig 3D).

To further verify such competition between Nb-015 and ACE2 observed in the structures, we subsequently conducted competitive binding assays based on ELISA and SPR, respectively. In the former experiment, the coated S-RBD was first incubated with ACE2 and then with the purified Nb-015-Fc protein (Nb-015 nanobody fused to human IgG1 Fc, S8 Fig). In the latter, S-RBD was immobilized on the CM5 chip and saturated Nb-015 was injected to S-RBD firstly, followed by the injection of ACE2. As expected, the results of both experiments showed that Nb-015 and ACE2 would compete against each other when bound to S-RBD (Fig 3E and 3F). We also aligned our structure to a previously reported cryo-electron microscopy structure of SARS-CoV-2 S-trimer in the pre-fusion conformation. It revealed that, similar to ACE2, Nb-015 could only bind to S-RBD in the up conformation (Fig 3G). Taken together, these results demonstrated that Nb-015 directly blocked the binding of SARS-CoV-2 to ACE2 for neutralization.

In light of the visibly reduced affinity of Nb-015 binding to Gamma S-RBD, we subsequently mapped the Gamma-specific mutations along the S-RBD sequence and found that the mutation of K417T was located in the epitope region (S7 Fig). Comparison of the S-RBD structures revealed that K417 in the WT S-RBD could well adjust its side chain to avoid any steric clash against Y101 of the nanobody. Residue T417 of the Gamma S-RBD, however, featured with a branched structure at the Cβ atom, which would cause some steric hindrance with Y101 and thereby compromise the interaction between the nanobody and S-RBD (S9A Fig). We also noticed that Beta S-RBD contained a K417N mutation at the same position. But such substitution apparently did not affect the binding. The asparagine residue possesses a branched structure at the Cγ rather than the Cβ atom. Accordingly, superimposition of a previously reported structure of Beta S-RBD with our structure showed that the side chain of N417 was adjusted similarly to K417 in WT S-RBD, thereby avoiding any steric conflict with the bound nanobody (S9A Fig).

Regarding the Omicron variants, we noted that Nb-015 possessed potent binding capacities towards BA.2 and BA.4/BA.5 S-RBDs but only showed a marginal binding towards XBB.1.5 and XBB.1.16 S-RBDs. We found that the mutation N460K, which was only identified in the XBB subvariants, would probably interfere with the Nb-015/S-RBD interactions (S7 Fig). This mutation would likely disrupt the two hydrogen bonds initially observed between S-RBD N460 and Nb-015 R99 and S100 (S9B Fig). In addition, the positively-charged residue K460 could generate charge repulsion with residue R99 of Nb-015, and the long side chain of lysine might also lead to some steric hindrance when binding to Nb-015, further compromising the binding.

### Structural basis for the neutralization by Nb-021

**Fig 4:**
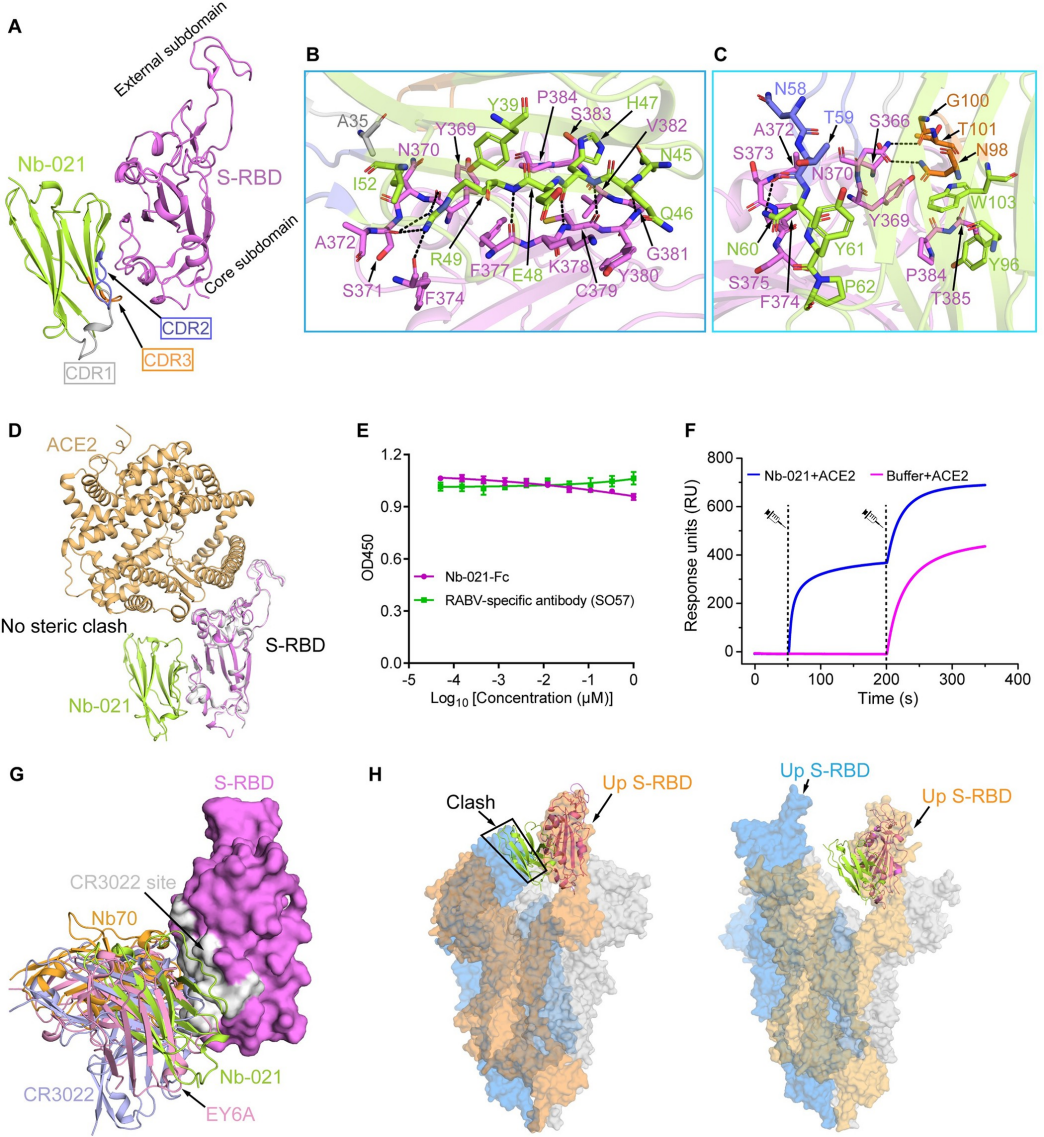
Structural basis for Nb-021 neutralization. **A,** Overall structure of Nb-021/S-RBD complex shown in cartoon. S-RBD is colored in magenta and Nb-021 in lemon. The CDR1, CDR2 and CDR3 of Nb-021 are indicated by arrows and colored in gray, light-blue and orange, respectively. The external subdomain and core subdomain of S-RBD are labelled. **B and C,** Detailed interactions between Nb-021 and SARS-CoV-2 S-RBD. **B,** Interactions between the residues 1–52 of Nb-021 and S-RBD. **C,** Interactions between the residues 53–113 of Nb-021 and S-RBD. Residues providing ≥2 van der Waals contacts are shown, the distance cutoff is 4.5 Å. Hydrogen bonds between Nb-021 and S-RBD (the distance cutoff is 3.1 Å) are presented as dashed lines. **D,** Structural alignment of Nb-021/S-RBD and ACE2/S-RBD complexes [PDB: 6M0J] [2]. There is no steric clash between Nb-021 and ACE2. **E,** Competitive ELISA-binding assay of Nb-021 and ACE2 to SARS-CoV-2 S-RBD. Error bar represents the mean ± SD of experiments performed in triplicates. **F,** SPR kinetics of the competitive binding of Nb-021 and ACE2 to SARS-CoV-2 S-RBD. The real-time binding profiles are recorded. **G,** Superimposition of the structures for Nb-021/S-RBD, Nb70/S-RBD [PDB code: 7X2K] [27], CR3022/S-RBD [PDB code: 6YM0] [25] and EY6A/S-RBD [PDB code: 6ZCZ] [26] complexes. Nanobodies Nb-021 and Nb70, antibodies CR3022 and EY6A are shown in cartoon and colored in lemon, orange, light-blue and pink, respectively. S-RBD is depicted as magenta surface and the previously identified CR3022 site on RBD is represented as gray. **H,** Superimposition of the Nb-021/S-RBD structure (shown in cartoon) onto the SARS-CoV-2 S-trimer structure with one-RBD-up conformation [the left panel, PDB code: 6VYB] [72] or two-RBD-up conformation [the right panel, PDB code: 7VND] [73]. The up conformation of the RBD in the S trimer structure is highlighted.

In parallel with the determination of Nb-015/S-RBD structure, we also successfully crystallized the protein complex of Nb-021 bound to SARS-CoV-2 S-RBD. The structure was subsequently solved at 2.4-Å resolution, and the final model was refined to *R*_work_ = 0.226 and *R*_free_ = 0.246, respectively (S1 Table). In our structure, each asymmetric unit of the crystal contains two copies of S-RBD/Nb-021 complex bound in a 1:1 binding mode, which are essentially identical with an RMSD of ~0.3 Å over 308 Cα atoms. Overall, Nb-021 targeted to a cryptic conserved epitope, distal from the receptor-binding site, on the core subdomain of S-RBD (Fig 4A). Detailed analyses revealed that the epitope was composed of S-RBD residues S366, Y369-S375 and F377-T385 and that the paratope contained Nb-021 residues A35, Y39, N45-R49, I52, N58-P62, Y96, N98, G100-T101 and W103. These amino acids formed an extended interaction network along the epitope/paratope interface, featuring with hundreds of inter-chain contacts and multiple strong inter-molecule hydrogen bonds (Fig 4B and 4C). It is notable that the identified paratope residues in Nb-021 are largely located in the nanobody framework region. These frame-residues involved in S-RBD binding include Y39, N45-R49, I52, N60-P62, Y96 and W103, providing 260 van der Waals (vdw) contacts (S2 Table). By contrast, the three CDRs only provide a rather limited contribution to the antigen recognition, with 33 vdw contacts (S2 Table). Such paratope features make Nb-021 a unique nanobody since almost all the antibodies and nanobodies would recognize their target antigens via the CDR loops.

To explore the structural basis of virus neutralization by Nb-021, we firstly superimposed our S-RBD/Nb-021 structure with the reported structure of ACE2 bound to S-RBD. The alignment showed that there is no steric clash between Nb-021 and ACE2, indicating that the nanobody would not interfere with the viral receptor binding (Fig 4D). Echoing the structural observation, the results of our competitive ELISA and SPR assays both showed that Nb-021 and Nb-021-Fc (Nb-021 nanobody fused to human IgG1 Fc, S8 Fig) did not affect the binding of ACE2 to S-RBD (Fig 4E and 4F). We further compared the binding site of Nb-021 on S-RBD with the identified epitopes away from ACE2 receptor-binding site. Structural superimposition revealed that the epitope of Nb-021 on S-RBD highly overlapped with antibodies-CR3022 and EY6A, and nanobody-Nb70 (Fig 4G). In addition, when we superimposed the Nb-021/S-RBD complex onto the S-trimer in prefusion conformation, we could find that, the binding of Nb-021 to S-trimer required at least two RBDs in the up conformation, which is also similar to that of CR3022, EY6A and Nb70 (Fig 4H). The binding of these antibodies and nanobody are demonstrated to lock the S-trimer in RBD-up conformation, further destabilize it and promote S1 shedding, leading to the premature transition into post-fusion conformation, thus inhibiting the virus entry [25–27]. Therefore, we propose that, similar to CR3022, EY6A antibodies and Nb70 nanobody, the neutralizing mechanism of Nb-021 is to destabilize the pre-fusion conformation of the trimeric spike.

In contrast to potent neutralization of WT and other variant viruses by Nb-021, the efficacy of the nanobody to neutralize the Omicron subvariants were dramatically compromised. We found that three mutations in the Omicron variants occurred in the Nb-021 epitope on S-RBD (S7 Fig). The three mutations, S371F, S373P and S375F, are clustered at a continuous loop, causing a main-chain conformational shift to residues S366 to K378 (S9C Fig). Such structural change would disrupt the interaction network (including multiple vdw contacts and hydrogen bonds) initially formed between residues S366, Y369-S375 and F377-K378 in S-RBD and amino acids A35, Y39, Q46-R49, I52, N58-P62, N98, G100-T101 and W103 in Nb-021, therefore resulting in decreased S-RBD binding and compromised neutralization against Omicron variants (Fig 4B and 4C, and S2 Table).

### Tandem-fusion of Nb-015 and -021 into a bispecific nanobody with improved efficacy against known SARS-CoV-2 variants

**Fig 5:**
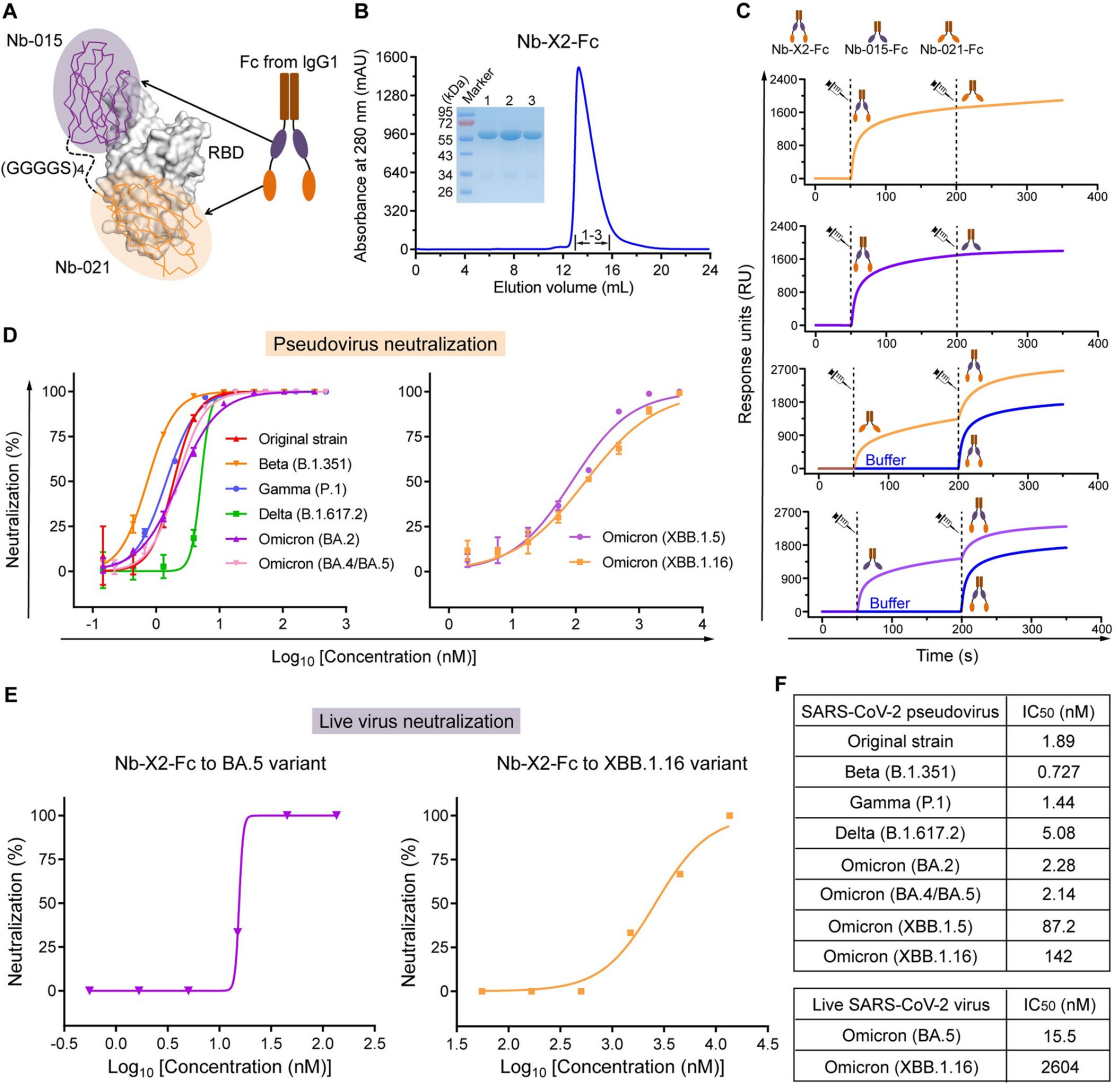
Bio-engineering of Nb-X2-Fc with improved neutralizing potency. **A,** Diagram showing the modification of Nb-X2-Fc. Nb-021 and Nb-015 are tandemly linked with a (GGGGS)_4_ linker and then fused to Human IgG1 Fc tag. S-RBD is shown in gray surface. Nb-021 and Nb-015 are depicted in ribbon mode and highlighted in orange and purple, respectively. **B,** Purification of Nb-X2-Fc by gel filtration chromatography. The inset figure shows the SDS-PAGE analyses of the indicated samples. **C,** Competitive SPR assays demonstrate that the two non-overlapping epitopes on S-RBD could be simultaneously bound by Nb-X2-Fc. The immobilized S-RBD was successively incubated with indicated proteins (Nb-015-Fc, Nb-021-Fc or Nb-X2-Fc). The real-time binding curves are shown. **D,** Pseudovirus-entry-inhibition assays using Nb-X2-Fc protein at the indicated concentrations. The mean ± SD represents error bar and the experiment was performed in triplicates. **E,** Neutralizing potency of Nb-X2-Fc against the indicated live virus of SARS-CoV-2. **F,** A table summarizing the IC_50_ values of Nb-X2-Fc against the indicated SARS-CoV-2 pseudovirus or live-virus.

It is known that nanobody multimerization could efficiently improve the efficacy, we therefore first characterized the neutralizing activity of nanobody-Fc (Nb-015-Fc and Nb-021-Fc) against pseudotyped SARS-CoV-2. To facilitate its comparison with the monovalent nanobodies, the molar concentration of the nanobody-Fc protein was calculated based on the molecular weight of the single molecule rather than the molecular weight of the nanobody-Fc dimer. Both Nb-015-Fc and Nb-021-Fc displayed significantly improved neutralizing activities. Nb-015-Fc displayed about 28~465-fold enhancement in the neutralizing activity, and Nb-021-Fc showed approximately 3~733-fold increase in the neutralization efficacy (S10 Fig and S3 Table). However, the two bivalent nanobodies only showed marginal neutralizing efficacy against the XBB.1.5 and XBB.1.16 viruses (S10 Fig). To broaden the neutralizing capacity of our nanobodies against the Omicron XBB variants, we then designed a bispecific nanobody conjugate (named Nb-X2-Fc) by tandem fusion of Nb-021, Nb-015 and IgG1 Fc (Fig 5A), aiming to break the mutational barrier observed for the individual nanobodies. Subsequently, Nb-X2-Fc was expressed in insect cells and prepared to high homogeneity and purity (Fig 5B). The competitive SPR assays showed that pre-bound Nb-X2-Fc could simultaneously inhibit the binding of both Nb-015 and Nb-021 to S-RBD. In contrast, with pre-bound Nb-015 or Nb-021, Nb-X2-Fc was still able to bind to S-RBD (Fig 5C). The results therefore demonstrated that the two non-overlapping epitopes on S-RBD could be readily recognized and simultaneously bound by Nb-X2-Fc (Fig 5C).

To explore the efficacy of the engineered nanobody conjugate, we then performed SPR assay to characterize the binding capacity of Nb-X2-Fc to XBB S-RBDs. Compared with the monovalent Nb-015 and Nb-021 nanobodies, Nb-X2-Fc expectedly showed a remarkable enhancement in the binding affinity towards XBB.1.5 and XBB.1.16 S-RBDs, with *K*_D_ values being calculated to be 2.81 nM and 3.64 nM, respectively (S11 Fig). We further performed pseudotyped and live virus entry-inhibition assays to evaluate the neutralizing breadth and efficacy of Nb-X2-Fc (Fig 5D and 5E). For pseudovirus neutralization, the final IC_50_ values were calculated to be 1.89, 0.727, 1.44, 5.08, 2.28 and 2.14 nM against the prototype virus and the Beta, Gamma, Delta, BA.2 and BA.4/BA.5 viruses, respectively (Fig 5F). Regarding XBB subvariants, Nb-X2-Fc showed weaker neutralizing activity, with IC_50_ values being determined to be 87.2 and 142 nM against XBB.1.5 and XBB.1.16, respectively (Fig 5F). Echoing the results of pseudovirus entry-inhibition assay, Nb-X2-Fc displayed potent neutralizing activity towards live BA.5 virus, showing an IC_50_ of 15.5 nM. Nb-X2-Fc also exhibited moderate neutralizing efficacy towards live XBB.1.16 virus, with the IC_50_ value being calculated to be 2.60 μM (Fig 5F). It’s noteworthy that, compared with Nb-015-Fc and Nb-021-Fc, Nb-X2-Fc showed a significantly improved neutralizing activity against the XBB subvariants. Taken together, the entry of all the viruses tested could be efficiently inhibited by the nanobody conjugate. These data demonstrated that Nb-X2-Fc could broadly neutralize SARS-CoV-2 variants with dramatically improved potency.

## Discussion

The ongoing SARS-CoV-2 pandemic remains a worldwide public health concern. Several therapeutic antibodies targeting SARS-CoV-2 S-RBD have been approved for clinical use [28–30]. However, the efficacy of most approved antibodies is significantly compromised when targeting the currently circulating Omicron variants [31,32]. Thus, the development of broad-spectrum and effective agents to combat various SARS-CoV-2 variants (especially Omicron) remains an urgent issue. Compared with other functional elements in S protein, S-RBD has become a worst-hit region for mutational escape [33], which carries 22 residue substitutions in XBB.1.5 and XBB.1.16 subvariants. In this case, re-screening antibodies to target the variant-specific S-RBD would require much time and effort. Moreover, the emergence of new escaped variants would necessitate for another round of screening, thereby significantly augmenting the workload and complexity involved in this endeavor. Therefore, developing strategies to overcome viral escape is still of great significance in SARS-CoV-2 prevention and control.

One simple strategy is to directly target the well-conserved epitopes on the S protein (e.g., the fusion peptide and the stem helix regions within the S2 subunit), for antibody screening [33]. However, it should be noted that, despite exhibiting broad-spectrum activity, the overall efficacies of S2-targeting antibodies are largely inferior to the S-RBD-targeting antibodies [34]. Another feasible strategy to resist viral escape is to multimerize the antibodies, using homologous or heterologous manners alone or in combination [35,36]. Multivalent engineering, under the dual effects of avidity and synergy, is expected to significantly enhance the antibody’s affinity, potency, and breadth towards targets [35,36]. However, since the light and heavy chains of traditional antibody both contribute to antigen binding, the antibody engineering is associated with certain complexity. Nanobody, with characteristics of small size (containing only one immunoglobulin domain), robust antigen-binding ability and excellent stability, represents an attractive option for therapeutic applications and a perfect template for multimeric strategy explorations [37,38].

Based on that, our study mainly focused on the nanobody identification, neutralization, action, and modification to finally obtain a broad-spectrum nanobody conjugate and explore the nanobody multimeric strategy. We identified two potent nanobodies, Nb-015 and Nb-021, that target two non-overlapping epitopes on SARS-CoV-2 S-RBD. The two nanobodies both possess efficient neutralization capacity against diverse VOCs and VOIs. The complex structures of the two nanobodies bound to S-RBD reveal that Nb-015 inhibits virus entry by competing against ACE2 binding and that Nb-021 neutralizes viral infection by destabilizing the pre-fusion conformation of the S protein. Structure-based engineering of the two nanobodies into a biparatopic nanobody conjugate (Nb-X2-Fc) could further increase the neutralization breadth and improve the inhibition efficacy against known SARS-CoV-2 variants, including the latest Omicron XBB.1.5 and XBB.1.16 viruses.

There are some other multivalent molecules that have been developed during the SARS-CoV-2 pandemic. For example, Schoof et al., Koenig et al., or Xiao et al. identified trivalent therapeutics [mNb6-tri [39], VHH EEE [40] or ACE2_615_-foldon [41]], which showed excellent potency against pseudotyped SARS-CoV-2 virus (prototype), with IC_50_ values being calculated to be 0.12 nM, 0.52 nM and 0.59 μg/mL, respectively. While Rothenberger et al., Hunt et al., or Du et al. reported trispecific DARPin (ensovibep) [42], trimeric minibinder TRI2-2 [43] or hexavalent biparatopic 10D12^VH1^-11C12^VH2^-10D12^VH3^ [44], which also exhibited high efficacy towards pseudotyped viruses (prototype and circulating variants), with IC_50_ values being calculated to be 0.4–6.1 ng/mL, 0.025–0.733 nM and 0.020–0.104 nM, respectively. The IC_50_ values of these multivalent molecules are better than that of Nb-X2-Fc calculated in this study. However, as we previously reported, the disparity of the IC_50_ values calculated via the pseudovirus neutralization assay might partially due to the differences in the experimental conditions and/or the materials used in the pseudovirus-entry-inhibition assay [19].

In light of the excellent physicochemical properties and potent activity of nanobodies, multiple studies have been focusing on the development of nanobody drugs against SARS-CoV-2 [45–48]. In terms of administration route used in these studies, some have chosen injection, while others have opted for intranasal administration. Both routes have shown effective viral clearance and demonstrated good preventive and therapeutic effects. However, it should be noted that in prophylactic treatment, multiple repeated administrations are usually required, which may cause the generation of anti-drug antibodies, consequently compromising the antiviral efficacy. Using different sets of antibodies and/or nanobodies for multi-round administrations might largely address this challenge. While Nb-X2-Fc is able to effectively neutralize Omicron-included SARS-CoV-2 variants, the nanobody conjugate might be developed as a drug reserve for clinical treatment of SARS-CoV-2 related diseases when different antibodies/nanobodies are required. Although our bispecific nanobody conjugate has not been validated in animal studies, we still believe that this agent would be effective in the *in-vivo* assays, given its potent efficacy observed in the *in-vitro* virus-neutralization level assays.

As a commonly used strategy, tandem coupling of Fc domain with our nanobodies is expected to prolong the half-life of the fusion protein, allowing a sufficient time window for the conjugate to exert its effectiveness *in vivo* [49]. Recent studies have revealed the roles of Fc-mediated effector functions (e.g., antibody-dependent cellular cytotoxicity and antibody-dependent cellular phagocytosis) in adaptive immune responses and SARS-CoV-2 clearance [50–53]. Through Fc-Fc gamma receptor (FcγR) interaction and the subsequent recruitment of immune cells (e.g., natural killer cells, macrophages, monocytes, and neutrophils), the Fc-containing therapeutics (e.g., antibodies and ACE2-Fc protein) could enhance protective efficacy in SARS-CoV-2 infected animal models [50–53]. By analogy, we believe that the designed Nb-X2-Fc may also exhibit Fc-effector functions that contribute to *in-vivo* protective activity.

Structural analysis revealed that the footprint of Nb-015 on S-RBD is extensively overlapped with the previously identified RBS-A site. Antibodies targeting this site can exert neutralization by directly competing against ACE2 binding. Unfortunately, it has also been shown that antibodies bound at RBS-A could be easily compromised or even completely lose neutralizing activity because a majority of the mutations identified in SARS-CoV-2 variants, especially in the Omicron viruses, are accumulated directly in or in the proximity to this site [35]. It is therefore relatively difficult to identify RBS-A targeting antibodies with cross-variant neutralization activities. Nb-015 possesses a broad neutralizing capacity against both the WT and the Omicron (BA.2 and BA.4/BA.5)-included variant viruses. In light of these results, we believe that the epitope recognized by Nb-015 likely represents a promising target for the development of ACE2-competing biomacromolecules with pan-variant neutralization capacity against SARS-CoV-2.

Unlike Nb-015, Nb-021 binds to an epitope within the CR3022 site. Unlike RBS-A that is hyper-mutated, the number of CR3022-site mutations is rather limited during SARS-CoV-2 evolution. Accordingly, some antibodies targeting CR3022 site are shown to cross-neutralize multiple SARS-CoV-2 variants [54]. Nb-021 is also insensitive to most mutations on S-RBD and exhibits potent binding and neutralization against Alpha, Beta, Gamma, Delta, Kappa, Lambda and Mu variants. Regarding the Omicron viruses, Nb-021 displayed reduced binding towards S-RBDs due to the three important mutations within the CR3022 site (S371F/L, S373P and S375F). It is notable that Nb-021 retains three-digit nano-molar affinity for S-RBD of the Omicron (BA.2) variant, which is only about 3-fold lower than that for Nb-015 towards Gamma S-RBD. The neutralization efficacy of Nb-021 against Omicron (BA.2) variant, however, is more than 15-fold lower than that of Nb-015 against the Gamma virus. It should be noted that the CR3022 site is a ‘cryptic site’ that requires two or more S-RBDs in the up-conformation for antibody binding [36]. The F371/P373/F375 triple mutations in Omicron spike have been shown to stabilize the one-RBD-up conformation, excluding the binding of antibodies targeting CR3022 site [37]. The marked decrease in Omicron neutralization by Nb-021 therefore likely arises from two aspects: 1, decreased affinity towards S-RBD and 2, stabilized spike conformation excluding Nb-021 binding.

Moreover, the CR3022 site is also a highly conserved epitope across sarbecovirus (including SARS-CoV-2 and SARS-CoV viruses) [54]. However, Nb-021 could not cross-neutralize SARS-CoV infection (S12A and S12B Fig). Further amino-acid sequence alignment showed that three residues (A372, S373 and P384 in SARS-CoV-2, T359, F360 and A371 in SARS-CoV) in the binding epitope of Nb-021 on S-RBD are different between SARS-CoV and SARS-CoV-2 (S12C Fig). We believe the substitutions of these three amino acids would disrupt the binding of Nb-021 to SARS-CoV S-RBD.

Up to now, several nanobodies targeting similar binding site to that of Nb-015 [e.g., aRBD-2 [46], 10D12 [44] and Sb14 [55]] or Nb-021 [e.g., VHH-72 [56], Nb70 [27], Nb30 [57], Nb34 [58], 1–8 [59], 2–31 [59], S43 [48], Sb68 [55] and F2 [60]] have been reported. Although these nanobodies recognize similar binding site, their paratope residues that interact with S-RBD are quite distinct (S13 Fig). It is notable that, unlike these reported nanobodies, Nb-021 binds S-RBD mainly via amino acids in the nanobody framework region, rather than by using residues in the CDR loops. In light of this unique antigen-recognition feature, we propose that Nb-021 might serve as a framework scaffold for the development of a single-molecule nanobody capable of recognizing two epitopes in S-RBD (e.g., by grafting CDRs from other RBD-recognizing antibodies or nanobodies into Nb-021). This dual-targeted nanobody is expected to behave similarly to Nb-X2-Fc, featuring with broad breadth and high potency against SARS-CoV-2 variants by binding to two different epitopes in S-RBD.

## Materials and methods

### Alpaca immunization and VHH library preparation

The SARS-CoV-2 S-RBD specific nanobody repertoire was prepared by CHENGDU NB BIOLAB CO., LTD as previously described [19]. Briefly, one alpaca was primarily immunized with 0.5 mg recombinant S-RBD protein on day 0, and then boost immunized with 0.25 mg S-RBD on day 21 and 42, respectively. At day 49, ~50 mL blood was collected for the isolation of peripheral blood mononuclear cells (PBMCs). Total RNA was extracted from the PBMCs and then reverse-transcribed into cDNA. The VHH genes were separated after nested PCR amplification and then cloned into pComb3XSS vector. For VHH libraries preparation, the recombinant vectors were electroporated into TG1 competent cells, and individual phage clones were next recovered after two rounds of panning.

### Gene construction and protein preparation

For nanobody preparation, the coding sequence for individual nanobody fused with a C-terminal 6×His tag (the residual His tag on nanobody is retained in all the subsequent experiments) was constructed into pNCMO2 vector and further electroporated into *Brevibacillus choshinensis* SP3 cells as previously reported [61]. After three-day expression, the cell culture supernatant was respectively collected for protein purification. Nanobodies were firstly eluted from Ni-TED NUPharose FF beads (NUPTEC) and then injected onto a Superdex 75 10/300 GL column (GE Healthcare) with the buffer containing 10 mM HEPES-NaOH (pH 7.5), 150 mM NaCl.

SARS-CoV-2 S-RBDs [the original strain (residues 320–537 in spike protein, GenBank accession number: MN908947.3), Alpha variant (B.1.1.7 lineage bearing N501Y mutation in S-RBD), Beta variant (B.1.351 lineage bearing K417N, E484K and N501Y mutations in S-RBD), Gamma variant (P.1 lineage bearing K417T, E484K and N501Y mutations in S-RBD), Delta variant (B.1.617.2 lineage bearing L452R and T478K mutations in S-RBD), Kappa variant (B.1.617.1 lineage bearing L452R and E484Q mutations in S-RBD), Lambda variant (C.37 lineage bearing L452Q and F490S mutations in S-RBD), Mu variant (B.1.621 lineage bearing R346K, E484K and N501Y mutations in S-RBD), Omicron variant (BA.2 lineage bearing G339D, S371F, S373P, S375F, T376A, D405N, R408S, K417N, N440K, S477N, T478K, E484A, Q493R, Q498R, N501Y and Y505H mutations in S-RBD; BA.4/BA.5 lineage bearing G339D, S371F, S373P, S375F, T376A, D405N, R408S, K417N, N440K, L452R, S477N, T478K, E484A, F486V, Q498R, N501Y and Y505H mutations in S-RBD; XBB.1.5 lineage bearing G339H, R346T, L368I, S371F, S373P, S375F, T376A, D405N, R408S, K417N, N440K, V445P, G446S, N460K, S477N, T478K, E484A, F486P, F490S, Q498R, N501Y and Y505H mutations in S-RBD; XBB.1.16 lineage bearing G339H, R346T, L368I, S371F, S373P, S375F, T376A, D405N, R408S, K417N, N440K, V445P, G446S, N460K, S477N, T478R, E484A, F486P, F490S, Q498R, N501Y and Y505H mutations in S-RBD)], SARS-CoV S-RBD (residues 307–523 in spike protein, GenBank accession number: AY278554.2), human ACE2 peptidase domain (PD) (residues 19–615, GenBank accession number: BAB40370.1) and Fc-fused proteins (Nb-015-Fc, Nb-021-Fc and Nb-X2-Fc) used for further experiments were expressed in *Spodoptera frugiperda* Sf9 cells by Bac-to-Bac baculovirus expression system (Invitrogen). For S-RBD production, a GP67 signal peptide, a Trx tag, a 6×His tag and an Enterokinase (EK) cleavage cite were fused in tandem manner at the N terminus of S-RBD coding sequence for protein secretion, folding, purification and tag removal. These gene fragments were then cloned into pFastBac1 vector, respectively. The coding fragment of ACE2, which fused with a GP67 signal peptide at N terminus to facilitate protein secretion and a 6×His tag at C terminus for protein purification, was also inserted into pFastBac1 vector for protein preparation. The recombinant plasmids encoding Fc-fused proteins was individually constructed by inserting the coding sequences for Nb-015 or Nb-021 or Nb-X2 [Nb-021-(GGGGS)_4_-Nb-015, a tandem linkage of Nb-021 and Nb-015 by a flexible (GGGGS)_4_ linker] into the pFastBac1 vector which had been modified with a N-terminal GP67 signal peptide and a C-terminal human IgG1 Fc fragment. Bacmid transfection, virus amplification and protein production were all conducted with Sf9 cells. Cell culture supernatants were collected at 72 hours.

For S-RBDs, the proteins were initially purified from the cell culture supernatants by a 5-mL His-Trap excel column (GE Healthcare) and then treated by EK protease (a gift from the laboratory of Li Yang, Sichuan University) to remove the N-terminal Trx and His tags. Further purification was conducted with Superdex 200 Increase 10/300 GL column (GE Healthcare) in 10 mM HEPES-NaOH (pH 7.5) and 150 mM NaCl buffer.

For ACE2, the protein eluted from His-Trap was then purified via ion-exchange chromatography using a Source 15Q column (GE Healthcare). Finally, the protein was loaded on a Superdex 200 Increase 10/300 GL column with the final buffer containing 10 mM HEPES-NaOH (pH 7.5) and 150 mM NaCl.

The Fc-fusion proteins were initially purified by HiTrap rProtein A FF column (GE Healthcare) for affinity chromatography. The eluted proteins were then loaded onto a Superdex 200 Increase 10/300 GL column in the buffer containing 10 mM HEPES-NaOH (pH 7.5) and 150 mM NaCl. An antibody (SO57) targeting rabies virus (RABV) glycoprotein was prepared from HEK-293T cells and is used as a control for the competitive ELISA.

### ELISA

SARS-CoV-2 original strain S-RBD were diluted in 0.05 M carbonate-bicarbonate coating buffer (pH 9.6) and then coated (200 ng per well) on 96-well microtiter plates (Corning) at 4°C overnight. Subsequently, the wells were blocked with PBST containing 5% non-fat powdered milk (Sangon Biotech) for 1.5 hour at room temperature. For the binding between S-RBD and nanobodies, the indicated proteins (ACE2, Nb-011 to Nb-028) at single concentration (2 μg/mL, 100 μL per well) or three-fold serially-diluted concentrations (from 1 μM, 100 μL per well) were added to the wells and incubated for 1.5 hours at room temperature. Then, the HRP-conjugated anti-His antibody (Proteintech) were added into the wells and incubated for 1 hour. For the competitive binding experiment, ACE2 (0.1 μM, 100 μL per well) were firstly incubated with the S-RBD coated on the plate for 1.5 hours at room temperature, followed by the addition of Nb-015-Fc or Nb-021-Fc or the RABV-specific antibody SO57 (3-fold serially-diluted concentrations from 1 μM, 100 μL per well) and incubation for another 1.5 hours. The goat anti-human IgG-HRP antibody (Merck Millipore) was next added and incubated for 1 hour. In each step, the plates were washed with PBST for three times. After HRP-conjugated antibody incubation, TMB solution (Beyotime) was added to react with the HRP conjugates for about 5 minutes at 37°C. The reaction was stopped by 2 M HCl. Finally, the emission OD450 was monitored via a microplate reader (BioTek).

### Pseudovirus neutralization assay

To obtain SARS-CoV pseudovirus, the original gene for SARS-CoV spike (GenBank accession number AY278554.2) were synthesized at Convenience Biology to include a C-terminal flag tag coding sequence and then subcloned into the pCAGGS vector via the EcoR I and Bgl II restriction sites. The subsequent spike-expression plasmids were then co-transfected with a plasmid encoding an Env-defective, luciferase-expressing HIV-1 genome (pNL4-3.luc.RE) into HEK-293T cells. Cell culture supernatant containing pseudotyped SARS-CoV was harvested 48 h post-transfection. Pseudotyped SARS-CoV-2 virus [the original strain, Alpha variant (B.1.1.7 lineage), Beta variant (B.1.351 lineage), Gamma variant (P.1 lineage), Delta variant (B.1.617.2 lineage), Kappa variant (B.1.617.1 lineage), Lambda variant (C.37 lineage), Mu variant (B.1.621 lineage), Omicron variant (BA.2 lineage), Omicron variant (BA.4/BA.5 lineage), Omicron variant (XBB.1.5 lineage), Omicron variant (XBB.1.16 lineage)] were purchased from Genomeditech (#GM-0220PV07, #GM-0220PV33, #GM-0220PV32, #GM-0220PV47, #GM-0220PV45, #GM-0220PV44, #GM-0220PV53, #GM-0220PV83, #GM-0220PV86, #GM-0220PV98, GM-0220PV106, GM-0220PV120, respectively).

For the pseudovirus neutralization assays, the HEK-293T cells that could stably express human ACE2 (HEK293T-ACE2) were initially seeded in 96-well cell-culture plates (Corning) with 1×10^4^ cells/well and cultured at 37°C. 3-fold serially-diluted concentrations of monovalent nanobodies or Fc-fused proteins were separately incubated with pseudotyped particles at 37°C for 60 minutes. Then, the protein/pseudovirus mixtures were added into HEK293T-ACE2 cells and replaced with fresh complete medium after 24 hours post-infection. After the next 48-hour incubation at 37°C, Luciferase activity in HEK293T-ACE2 cells was determined by One-Lumi II Firefly Luciferase Assay Kit according to the manufacturer’s instructions (Beyotime). The half maximal inhibitory concentration (IC_50_) values were calculated by GraphPad Prism 6.

### Live virus neutralization assay

Live SARS-CoV-2 virus (BA.5 or XBB.1.16) neutralization assays were performed based on the cytopathic effect (CPE) [62]. Vero E6 cells (5×10^4^) were seeded in 96-well plates and grown overnight at 37°C. 3-fold serially-diluted concentrations of Nb-X2-Fc was preincubated with the live virus at 37°C for 1 h, the protein/virus mixture was then added into Vero E6 cells. CPE were observed and recorded on day 3 post-infection. The IC_50_ values of Nb-X2-Fc were calculated using GraphPad Prism 6.

### Syncytium formation assay

The syncytium-formation inhibition activities of nanobodies (Nb-011 to Nb-028) were assessed by SARS-CoV-2 S protein-mediated cell-cell fusion assay. Initially, the plasmids encoding SARS-CoV-2 original strain S or EGFP were co-transfected into HEK-293T cells (HEK293T-S/EGFP cells) using Lipo8000 (Beyotime). After 40 hours post-transfection, the HEK293T-S/EGFP cells were seeded into 96-well cell-culture plates with 2.5×10^4^ cells/well and then mixed with the diluted ACE2 or monovalent nanobodies followed by the incubation at 37°C for 1 hour. Subsequently, HEK293T-ACE2 cells were seeded into the plates with 5×10^4^ cells/well and the mixture was co-incubated at 37°C for 4 hours. The final concentrations of the indicated proteins were used at 1 μM or 10 μM. The formation of syncytia was presented by fluorescence microscope (Olympus).

### SPR assay

All the SPR experiments were carried out with the BIAcore 8K and X100 system (GE Healthcare). For affinity measurements, purified S-RBDs were separately immobilized onto CM5 sensor chip (GE Healthcare) using the Amine Coupling Kit (GE Healthcare). Two-fold serially diluted concentrations of analytes (Nb-015 or Nb-021 or Nb-X2-Fc) were successively flowed over the sensor ship in the running buffer containing 10 mM HEPES-NaOH (pH 7.5), 150 mM NaCl and 0.05% Tween-20 at a rate of 30 μL/min. Following each cycle, the ligand was re-generated with pH 2.0 glycine. The final kinetic data were analyzed with Biacore Evaluation Software (GE Healthcare) and the dissociation constant (*K*_D_) was further calculated with 1:1 (Langmuir) binding model for the slow-on/slow-off data and steady-state affinity model for the fast-on/fast-off data, respectively. For the competitive binding assays, the original strain S-RBD was loaded onto CM5 sensor chip as described in the kinetics experiments. Then, the primary injection at saturated concentration was flowed over the chip with a rate of 30 μL/min for 150 s immediately followed by the second injection at the same rate for another 150 s. After each cycle, the ligand was re-generated with pH 1.5 glycine.

### Crystallization

For crystallization screening, the nanobody/S-RBD (Nb-015/S-RBD or Nb-021/S-RBD) complex was prepared. In brief, SARS-CoV-2 original strain S-RBD was mixed with nanobody at a molar ratio of 1:1.2 and then incubated at 4°C for 2 hours. The mixture was then loaded on a Superdex 200 Increase 10/300 GL column with the buffer containing 20 mM Tris-HCl (pH 8.0), 150 mM NaCl to remove the unbound nanobody. Fractions containing the complex were then pooled and concentrated to 10 mg/mL. The crystallization screenings were performed using sitting-drop vapor diffusion method with multiple commercial crystallization kits (Hampton Research and Molecular Dimensions). In detail, 1 μL nanobody/S-RBD complex was mixed with 1 μL reservoir solution and then equilibrated against 70 μL reservoir solution at 18°C. The crystals for the Nb-015/S-RBD complex were grown in the condition consisting of 0.2 M Sodium formate (pH 7.2) and 20% w/v Polyethylene glycol 3350. And the crystals for Nb-021/S-RBD complex were obtained in conditions containing 0.1 M Sodium citrate tribasic dihydrate (pH 5.0) and 18% w/v Polyethylene glycol 20000.

### Data collection and structure determination

For data collection, the crystals were briefly soaked in reservoir solution mixed with 20% (v/v) glycerol and then flash-cooled into liquid nitrogen. X-ray diffraction data for Nb-015/S-RBD and Nb-021/S-RBD were collected at Shanghai Synchrotron Radiation Facility (SSRF) beamline BL19U1 and respectively processed with HKL3000 [63] and HKL2000 [64] for indexing, integration, and scaling. The structures of nanobody/S-RBD complex were determined by molecular replacement method using the Phaser program [65] from the CCP4 suite [66] with the previously reported SARS-CoV-2 RBD structure [PDB code: 6YZ5] [67] and nanobody structure [PDB 5TP3 [68] for Nb-015 and PDB 6B20 [69] for Nb-021] as the search models. The initial atomic models were further completed with Coot [70] and refined with phenix.refine in Phenix [71]. Final statistics for X-ray data collection and structure refinement are summarized in S1 Table. All structural figures were generated using PyMOL (https://pymol.org/).

## Supporting information

S1 Fig Amino-acid alignment of the 18 nanobodies with unique sequences identified in this study. The CDR regions are marked.
(TIF)

S2 Fig Solution behaviors of the other 16 nanobodies on Superdex 75 10/300 GL column. The inset figure shows the SDS-PAGE analyses of the indicated nanobodies.
(TIF)

S3 Fig Single-concentration ELISA-binding between 18 nanobodies and SARS-CoV-2 S-RBD. ACE2 was used as positive control. SARS-CoV-2 S-RBD was immobilized on 96-well plate and then incubated with nanobodies (purple) or ACE2 (blue). The emission OD450 was plotted as histograms. The error bar shows the mean ± SD of triplicates.
(TIF)

S4 Fig Inhibition of SARS-CoV-2 S-mediated syncytium-formation by the other 16 nanobodies at 10 μM concentration. The representative syncytia are marked with white arrows. Scale bar equals 100 μm.
(TIF)

S5 Fig Characterization of solution behaviors of SARS-CoV-2 S-RBDs by Superdex 200 Increase 10/300 GL column. The inset figure shows the SDS-PAGE analyses of the indicated S-RBDs.
(TIF)

S6 Fig Superimposition of the structures for Nb-015/S-RBD and CB6/S-RBD complexes [PDB code: 7C01] [74]. Nanobody Nb-015 and antibody CB6 are shown in cartoon and colored by cyan and orange, respectively. S-RBD is depicted as magenta surface and the RBS-A site on RBD is shown in gray.
(TIF)

S7 Fig Sequence profile highlighting the position of SARS-CoV-2 variant-specific mutations, the ACE2 footprint and the nanobody binding sites on S-RBD. The sequence of SARS-CoV-2 S-RBD is shown above the rectangle. Residue numbers are labelled every 10 amino acids above the sequence panel. The variant-specific S-RBD mutations, and the footprints of ACE2, Nb-015 and Nb-021 are individually highlighted with different colors.
(TIF)

S8 Fig Purification of Nb-015-Fc and Nb-021-Fc by gel filtration chromatography using Superdex 200 Increase 10/300 GL column. The inset figure shows the SDS-PAGE analyses of the indicated Fc-fusion proteins.
(TIF)

S9 Fig Potential mechanisms of the decreased efficacy of Nb-015 towards Gamma and XBB variants and Nb-021 towards Omicron variant. **A,** A magnified view of residue 417 in our S-RBD structure, in the Beta variant S-RBD structure [PDB code: 7NXA] [75] and in the Gamma variant S-RBD structure [PDB code: 7NXB] [75]. K417 of the WT strain, N417 of the Beta variant and T417 of the Gamma variant are shown as sticks and colored in magenta, yellow and lemon, respectively. Y101 in nanobody Nb-015 is depicted as orange sticks. The Cβ and Cγ atoms are highlighted with black arrows. **B,** A magnified view on the interactions between S-RBD N460 (magenta) and Nb-015 R99 and S100 (orange). The three residues are shown as sticks. Hydrogen bonds between Nb-015 and S-RBD (the distance cutoff is 3.1 Å) are presented as dashed lines. **C,** Alignment of the previously reported Omicron S-RBD structures to our complex structure of Nb-021 bound to S-RBD. The structures of Nb-021 bound to S-RBD, Omicron BA.2 S-RBD [PDB code: 7XB0] [76], Omicron BA.4/BA.5 S-RBD [PDB code: 7XWA] [77] and Omicron XBB.1 S-RBD [PDB code: 8IOV] [78] are colored in magenta, yellow, orange and cyan, respectively. The magnified picture shows the main-chain conformational change (residues S366-K378) in S-RBD.
(TIF)

S10 Fig Quantitative analysis of the neutralizing potency of Nb-015-Fc and Nb-021-Fc. **A-B,** Neutralizing potency of Nb-015-Fc (A) and Nb-021-Fc (B) characterized using SARS-CoV-2 pseudoviruses. Error bar stands for the mean ± SD. Experiments were performed in triplicates. **C,** A table summarizing the IC_50_ values of nanobody-Fc (Nb-015-Fc and Nb-021-Fc) against SARS-CoV-2 pseudoviruses.
(TIF)

S11 Fig SPR binding of Nb-X2-Fc to SARS-CoV-2 XBB subvariant S-RBDs. S-RBD from the indicated XBB subvariant was immobilized onto a sensor chip. Gradient concentrations of Nb-X2-Fc were then flowed over S-RBD on the chip surface. The obtained kinetic data were analyzed using the 1:1 binding model.
(TIF)

S12 Fig Nb-021 can not neutralize SARS-CoV. **A,** SPR analysis of the binding of Nb-021 to SARS-CoV S-RBD. **B,** Pseudovirus-entry-inhibition assay of Nb-021 against SARS-CoV. **C,** Amino-acid sequence alignment of SARS-CoV-2 S-RBD and SARS-CoV S-RBD. The footprint of Nb-021 on SARS-CoV-2 S-RBD is marked with blue triangles.
(TIF)

S13 Fig Amino-acid sequence alignment of the reported antibodies/nanobodies that recognize similar binding sites to those of our nanobodies. **A,** Sequence alignment of Nb-015, CB6 antibody (the heavy chain) and three nanobodies (aRBD-2, 10D12 and Sb14) that target similar binding site in S-RBD. The CDR regions are marked. **B,** Sequence alignment of Nb-021, CR3022 antibody (the heavy chain) and nine nanobodies (VHH-72, Nb70, Nb30, Nb34, 1–8, 2–31, S43, Sb68 and F2) that target similar binding site in S-RBD. The CDR regions are marked.
(TIF)

S1 Table Data collection and structure refinement statistics. (DOCX)

S2 Table Summary of the atomic binding details between Nb-021 and SARS-CoV-2 S-RBD. (DOCX)

S3 Table Summary of the IC_50_ values of mono-, bi-, and tetra-valent nanobodies against SARS-CoV-2 pseudoviruses. (DOCX)
