# Supplementary figures and images for: Development of a bispecific nanobody conjugate broadly neutralizes diverse SARS-CoV-2 variants and structural basis for its broad neutralization
Source: PLoS Pathog. 2023 Nov 30;19(11):e1011804. doi: 10.1371/journal.ppat.1011804 (PMC10688893; doi:10.1371/journal.ppat.1011804)

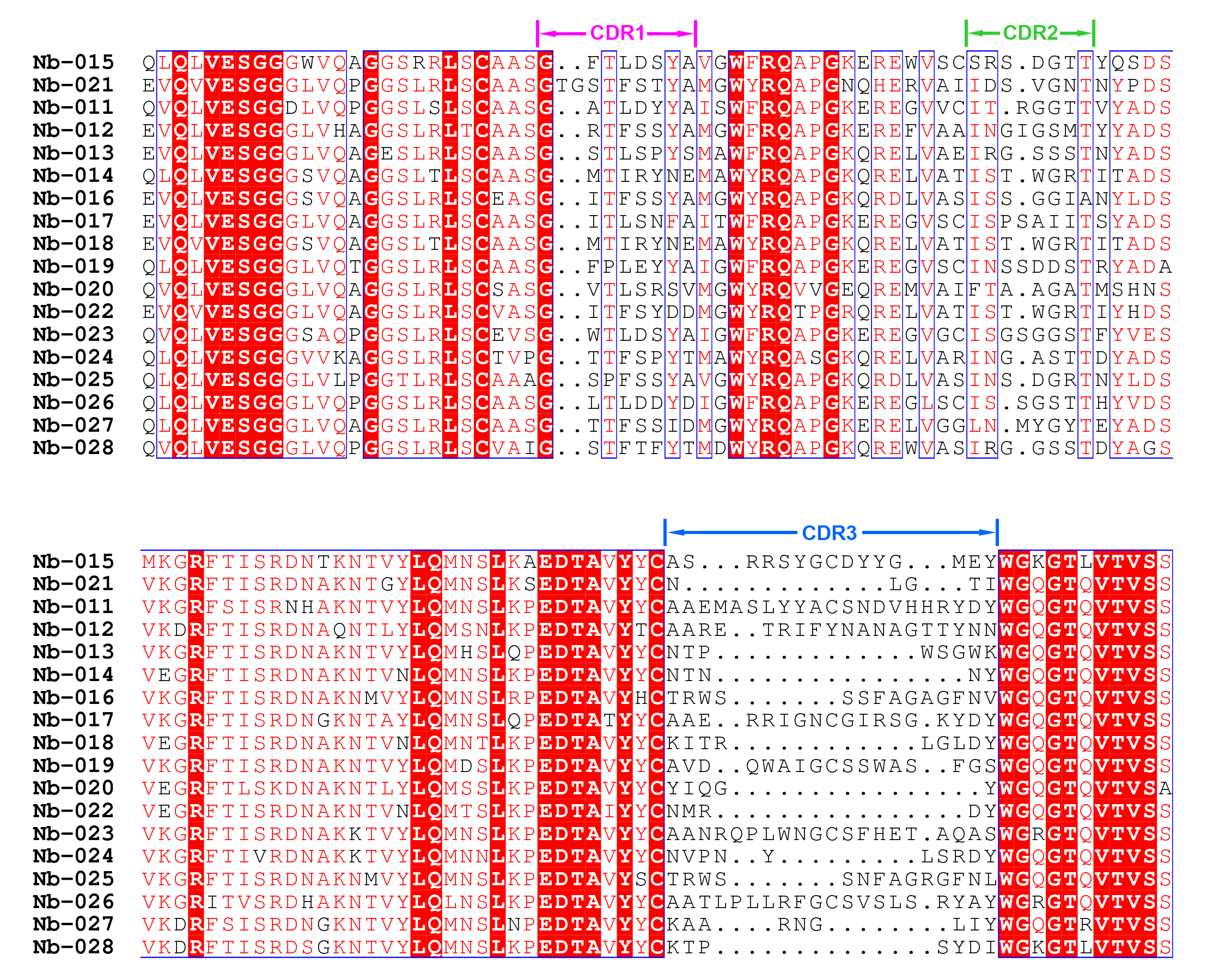

Supplement: S1 Fig — The CDR regions are marked. (TIF) [file ppat.1011804.s001.tif]

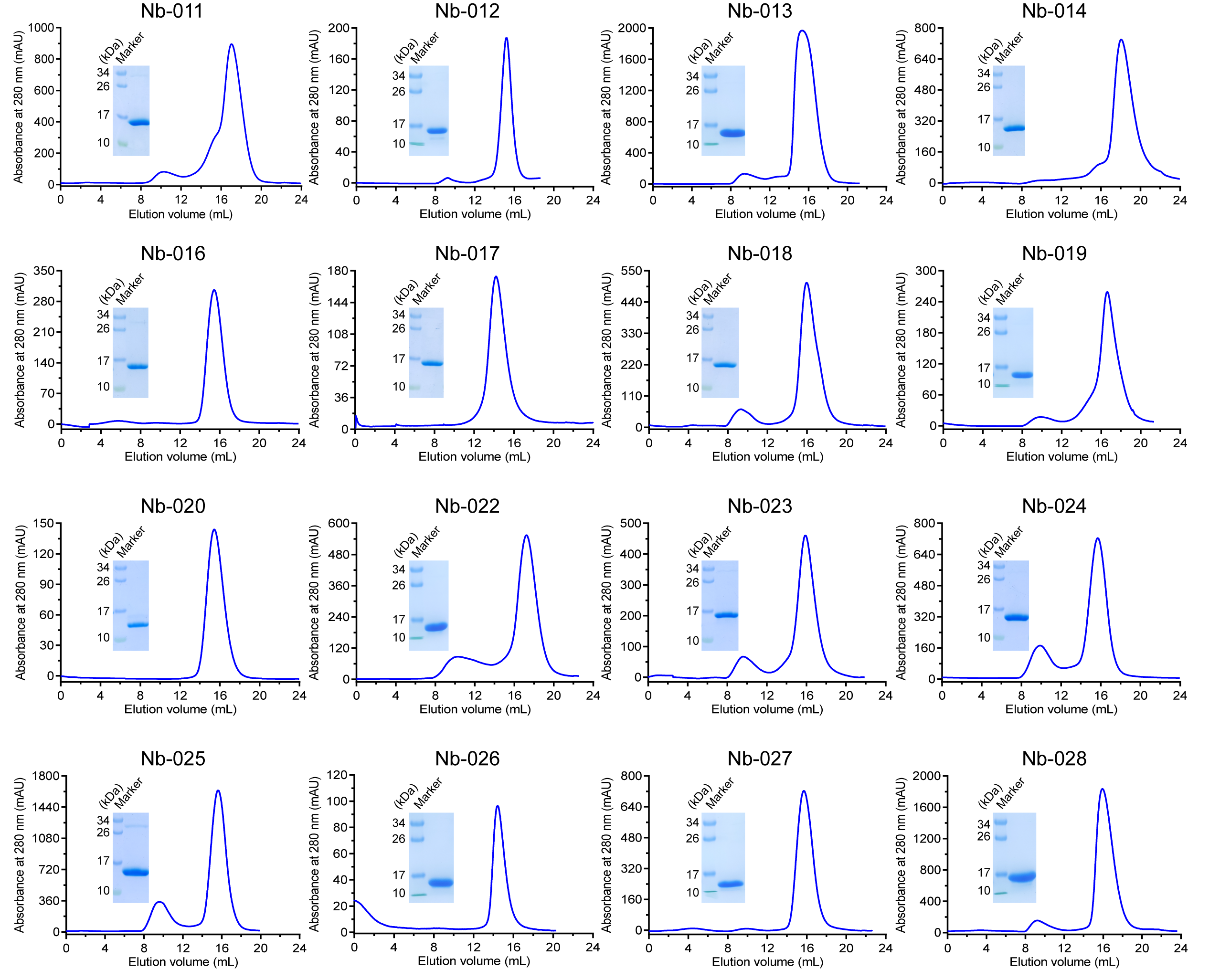

Supplement: S2 Fig — The inset figure shows the SDS-PAGE analyses of the indicated nanobodies. (TIF) [file ppat.1011804.s002.tif]

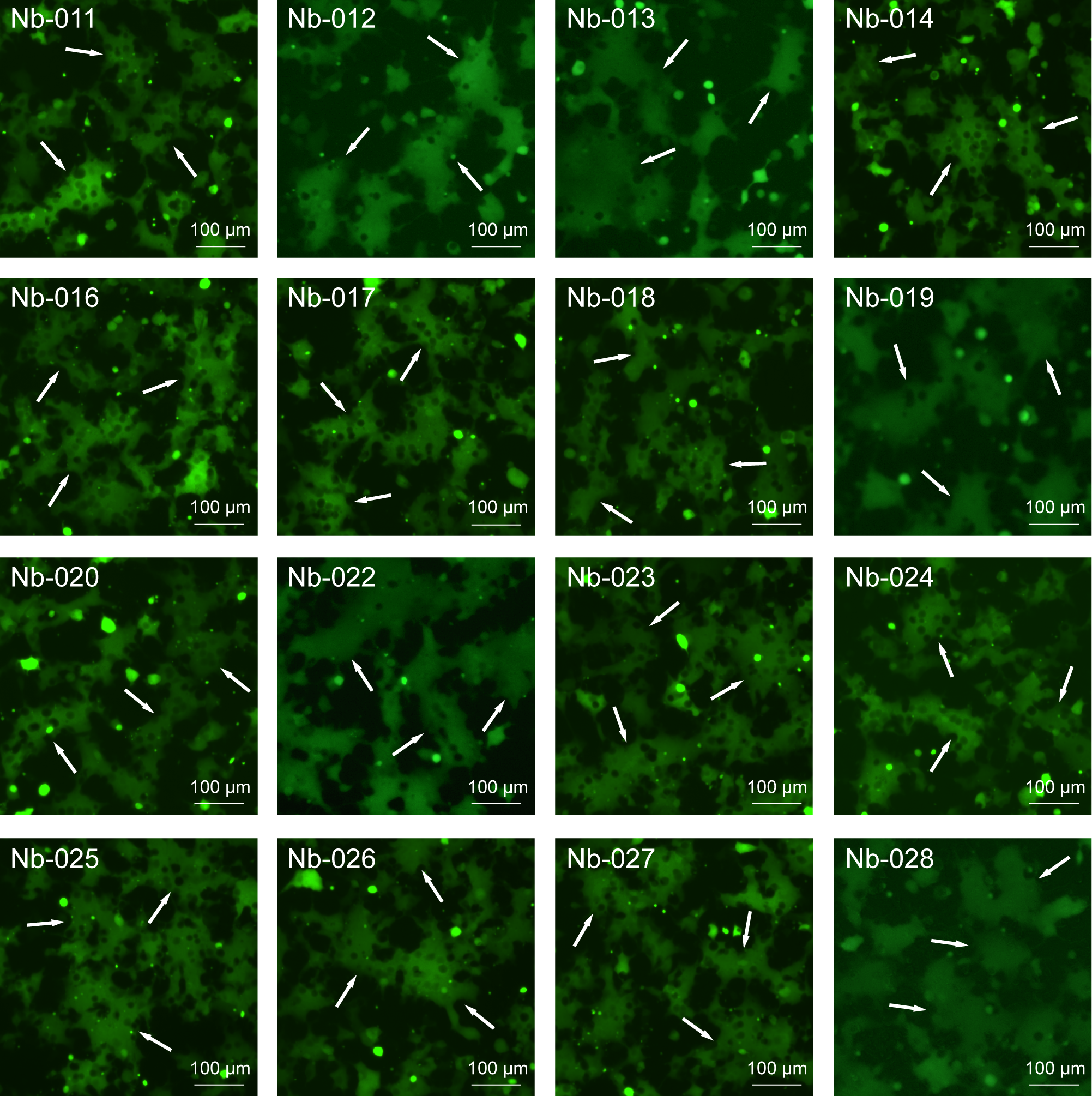

Supplement: S4 Fig — The representative syncytia are marked with white arrows. Scale bar equals 100 μm. (TIF) [file ppat.1011804.s004.tif]

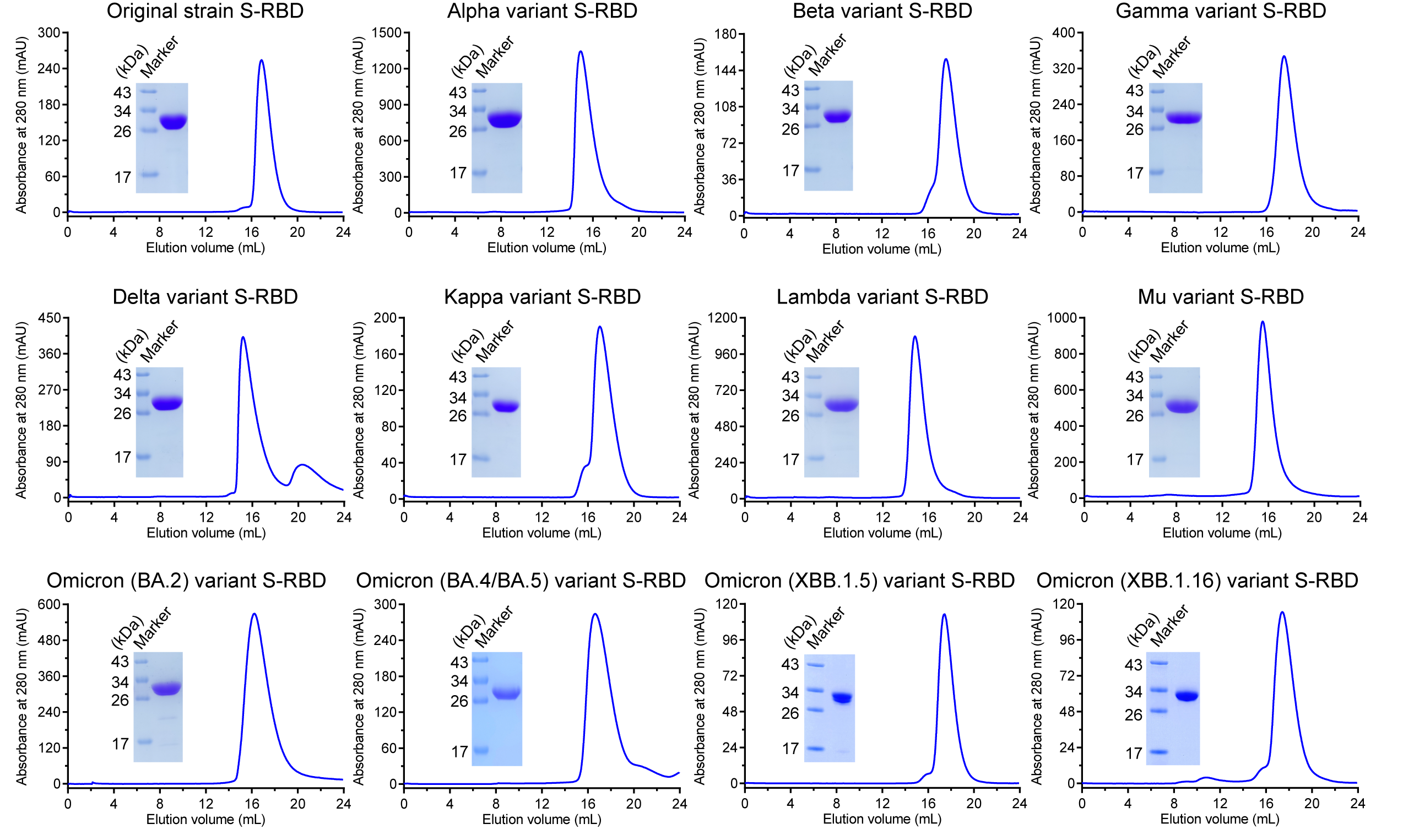

Supplement: S5 Fig — The inset figure shows the SDS-PAGE analyses of the indicated S-RBDs. (TIF) [file ppat.1011804.s005.tif]

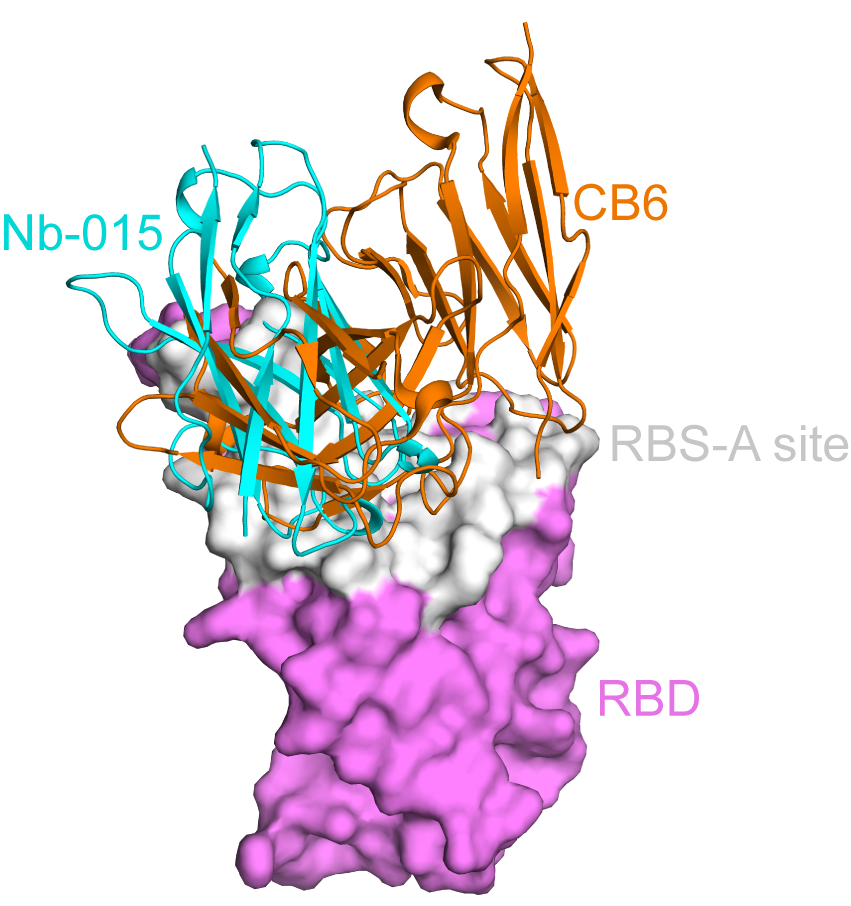

Supplement: S6 Fig — Nanobody Nb-015 and antibody CB6 are shown in cartoon and colored by cyan and orange, respectively. S-RBD is depicted as magenta surface and the RBS-A site on RBD is shown in gray. (TIF) [file ppat.1011804.s006.tif]

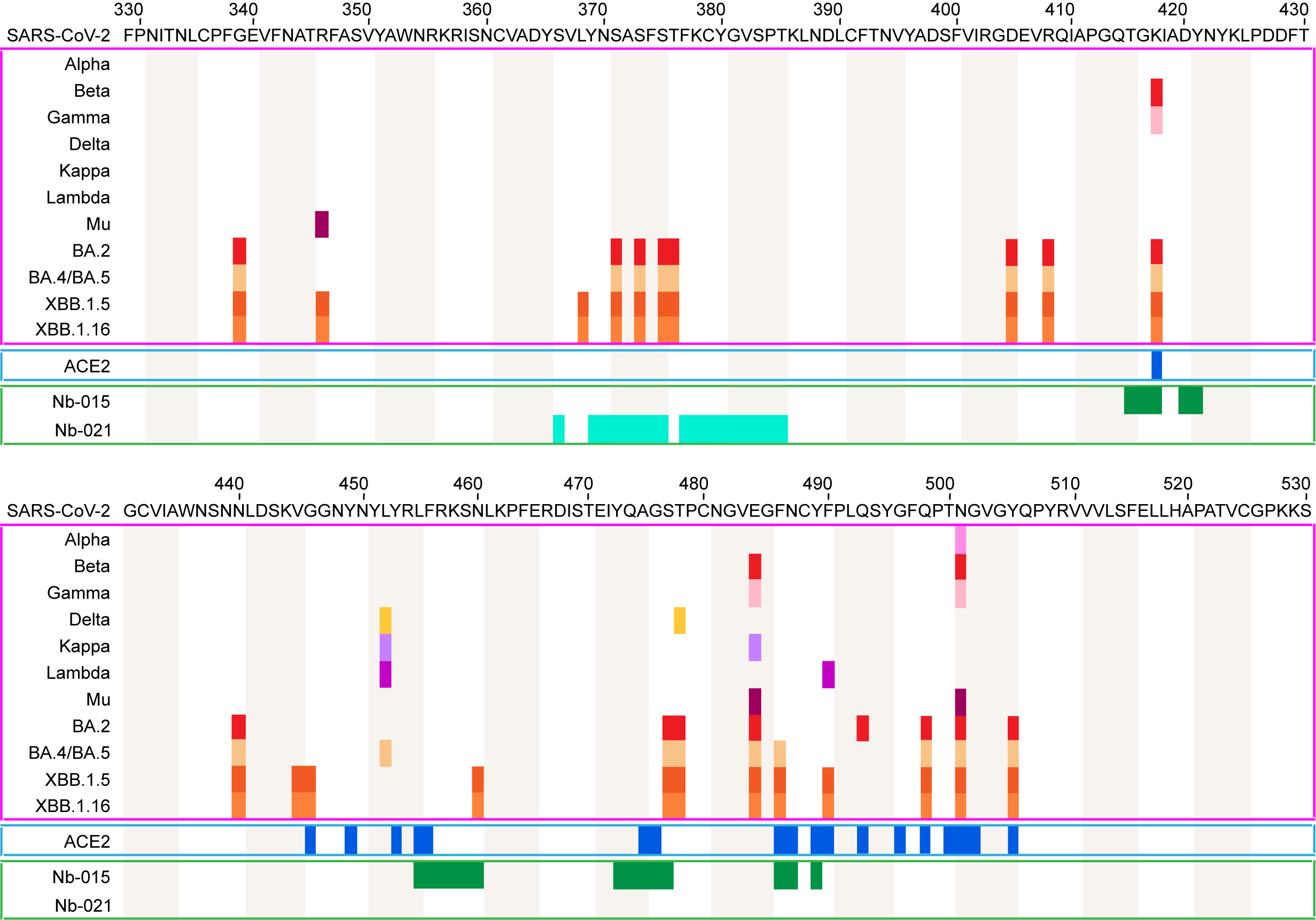

Supplement: S7 Fig — The sequence of SARS-CoV-2 S-RBD is shown above the rectangle. Residue numbers are labelled every 10 amino acids above the sequence panel. The variant-specific S-RBD mutations, and the footprints of ACE2, Nb-015 and Nb-021 are individually highlighted with different colors. (TIF) [file ppat.1011804.s007.tif]

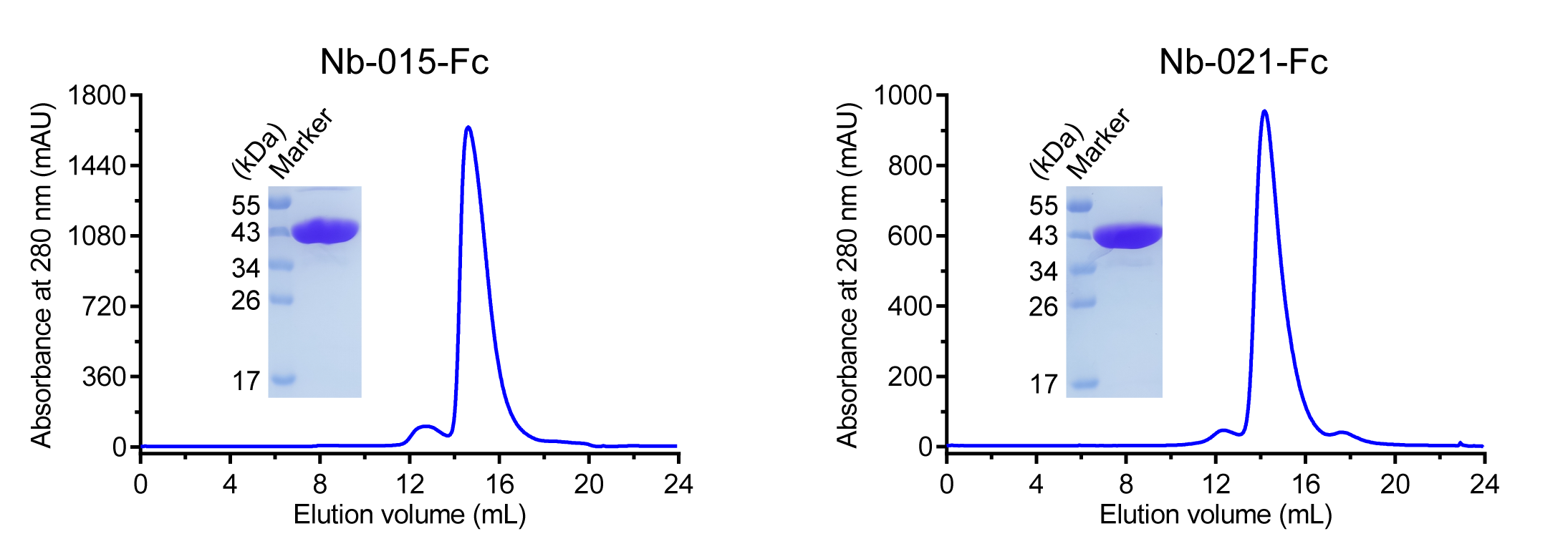

Supplement: S8 Fig — The inset figure shows the SDS-PAGE analyses of the indicated Fc-fusion proteins. (TIF) [file ppat.1011804.s008.tif]

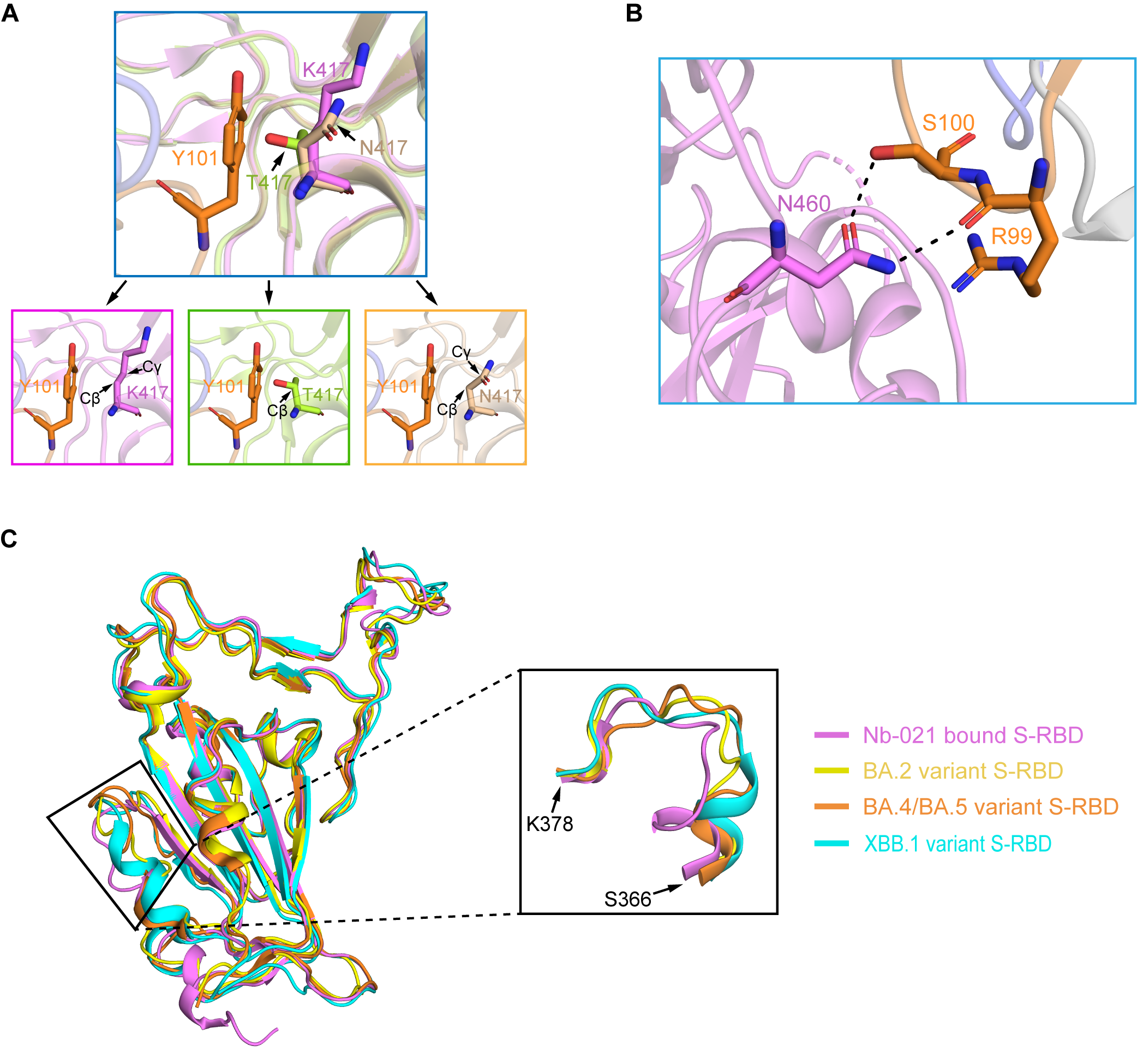

Supplement: S9 Fig — A, A magnified view of residue 417 in our S-RBD structure, in the Beta variant S-RBD structure [PDB code: 7NXA] [75] and in the Gamma variant S-RBD structure [PDB code: 7NXB] [75]. K417 of the WT strain, N417 of the Beta variant and T417 of the Gamma variant are shown as sticks and colored in magenta, yellow and lemon, respectively. Y101 in nanobody Nb-015 is depicted as orange sticks. The Cβ and Cγ atoms are highlighted with black arrows. B, A magnified view on the interactions between S-RBD N460 (magenta) and Nb-015 R99 and S100 (orange). The three residues are shown as sticks. Hydrogen bonds between Nb-015 and S-RBD (the distance cutoff is 3.1 Å) are presented as dashed lines. C, Alignment of the previously reported Omicron S-RBD structures to our complex structure of Nb-021 bound to S-RBD. The structures of Nb-021 bound to S-RBD, Omicron BA.2 S-RBD [PDB code: 7XB0] [76], Omicron BA.4/BA.5 S-RBD [PDB code: 7XWA] [77] and Omicron XBB.1 S-RBD [PDB code: 8IOV] [78] are colored in magenta, yellow, orange and cyan, respectively. The magnified picture shows the main-chain conformational change (residues S366-K378) in S-RBD. (TIF) [file ppat.1011804.s009.tif]

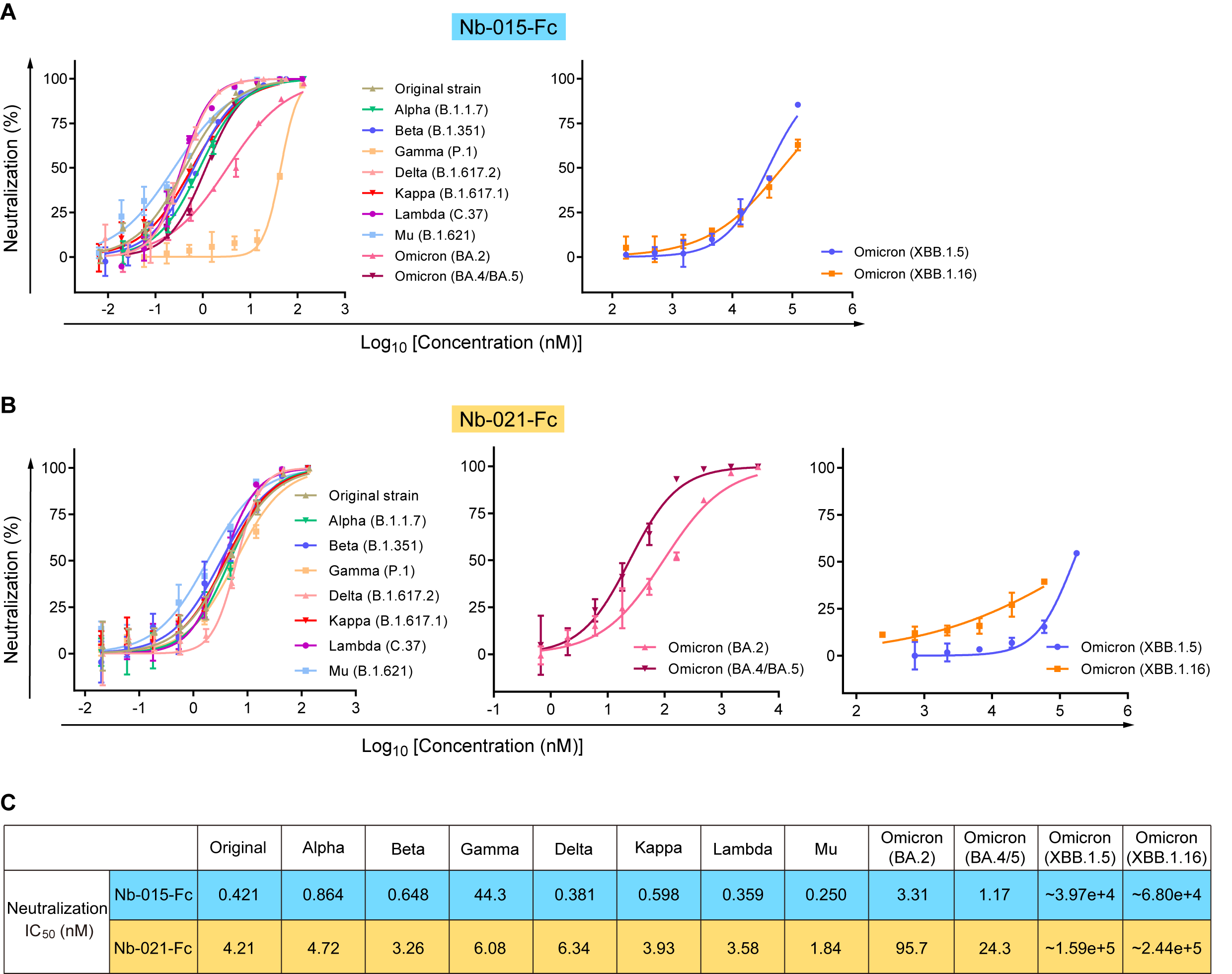

Supplement: S10 Fig — A-B, Neutralizing potency of Nb-015-Fc (A) and Nb-021-Fc (B) characterized using SARS-CoV-2 pseudoviruses. Error bar stands for the mean ± SD. Experiments were performed in triplicates. C, A table summarizing the IC50 values of nanobody-Fc (Nb-015-Fc and Nb-021-Fc) against SARS-CoV-2 pseudoviruses. (TIF) [file ppat.1011804.s010.tif]

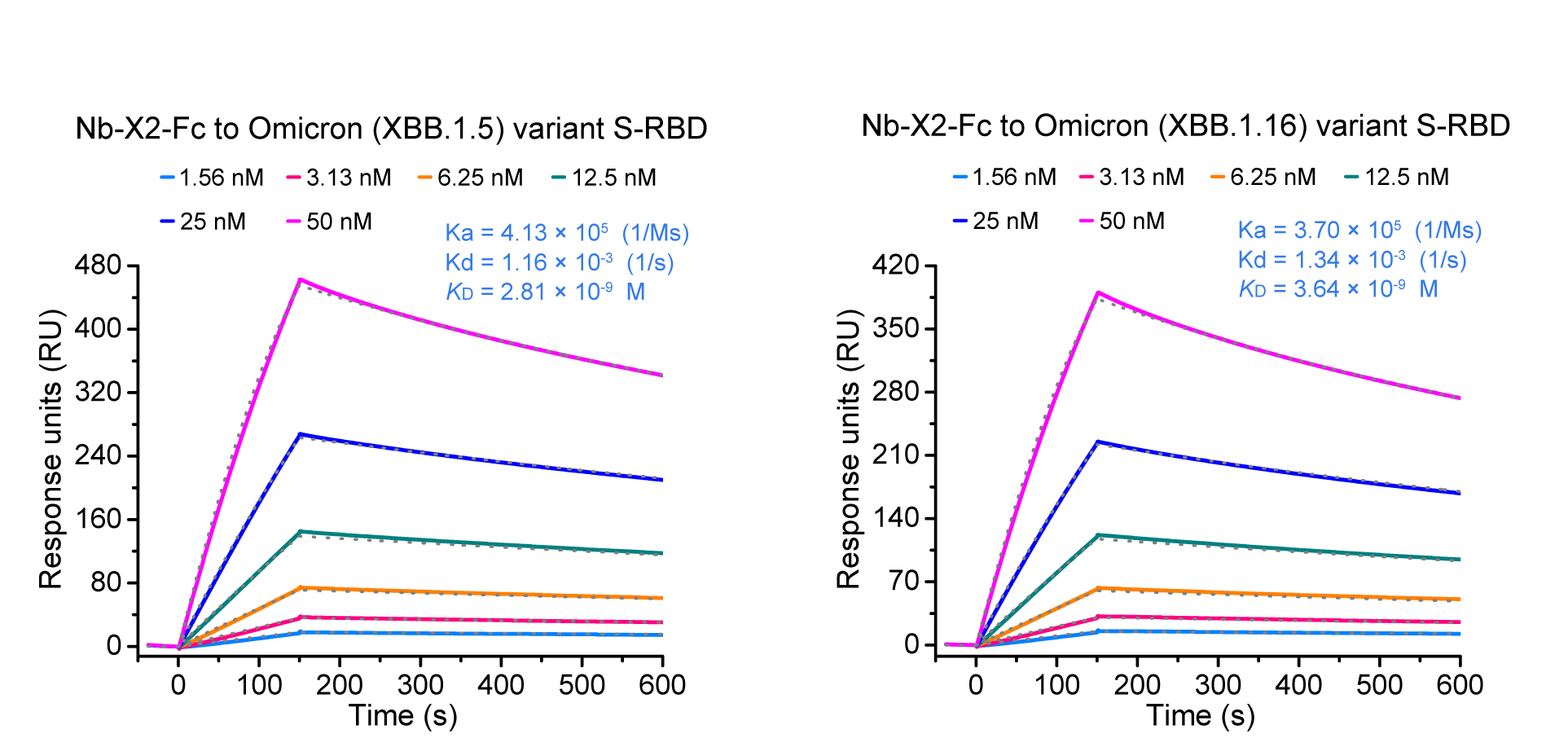

Supplement: S11 Fig — S-RBD from the indicated XBB subvariant was immobilized onto a sensor chip. Gradient concentrations of Nb-X2-Fc were then flowed over S-RBD on the chip surface. The obtained kinetic data were analyzed using the 1:1 binding model. (TIF) [file ppat.1011804.s011.tif]

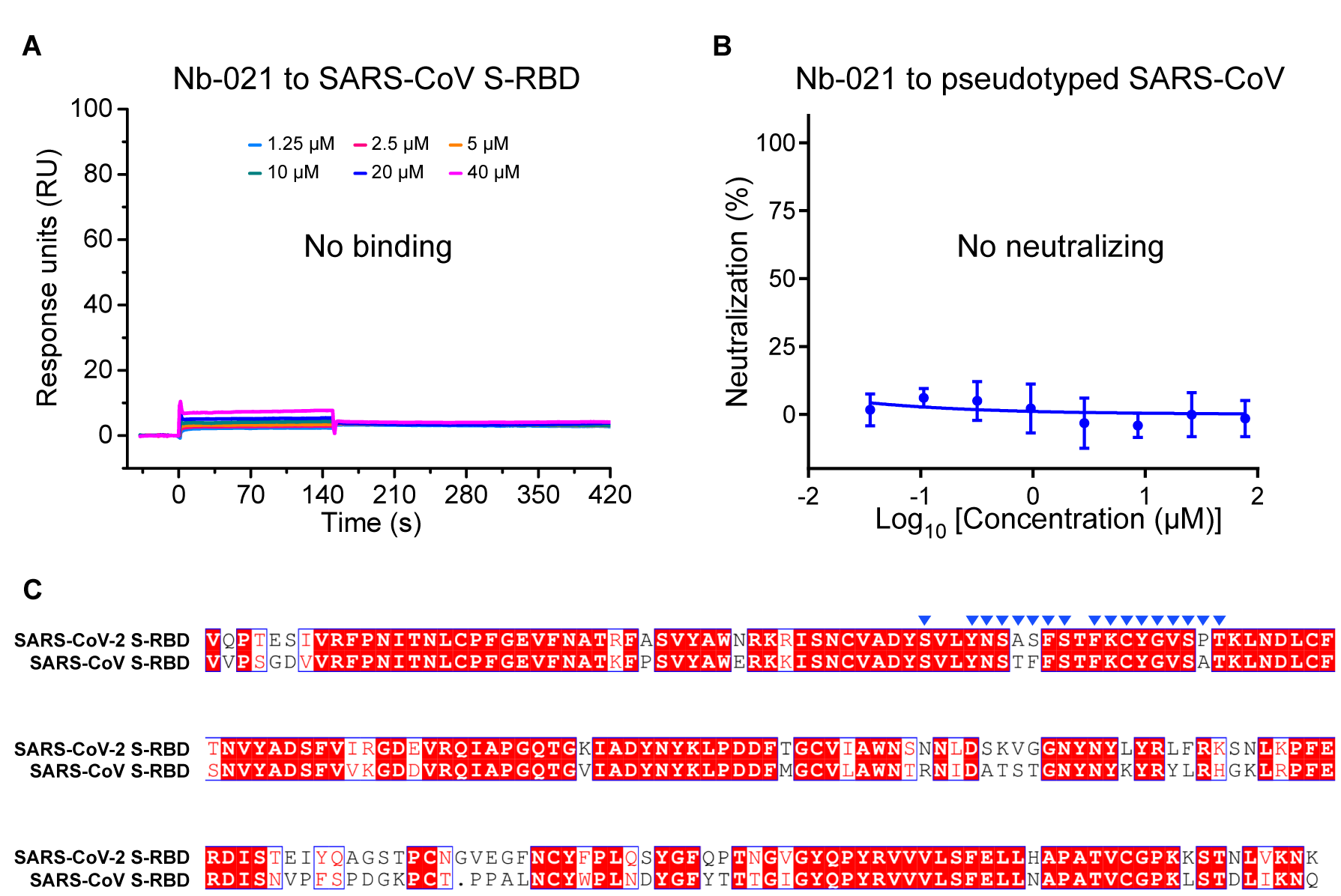

Supplement: S12 Fig — A, SPR analysis of the binding of Nb-021 to SARS-CoV S-RBD. B, Pseudovirus-entry-inhibition assay of Nb-021 against SARS-CoV. C, Amino-acid sequence alignment of SARS-CoV-2 S-RBD and SARS-CoV S-RBD. The footprint of Nb-021 on SARS-CoV-2 S-RBD is marked with blue triangles. (TIF) [file ppat.1011804.s012.tif]

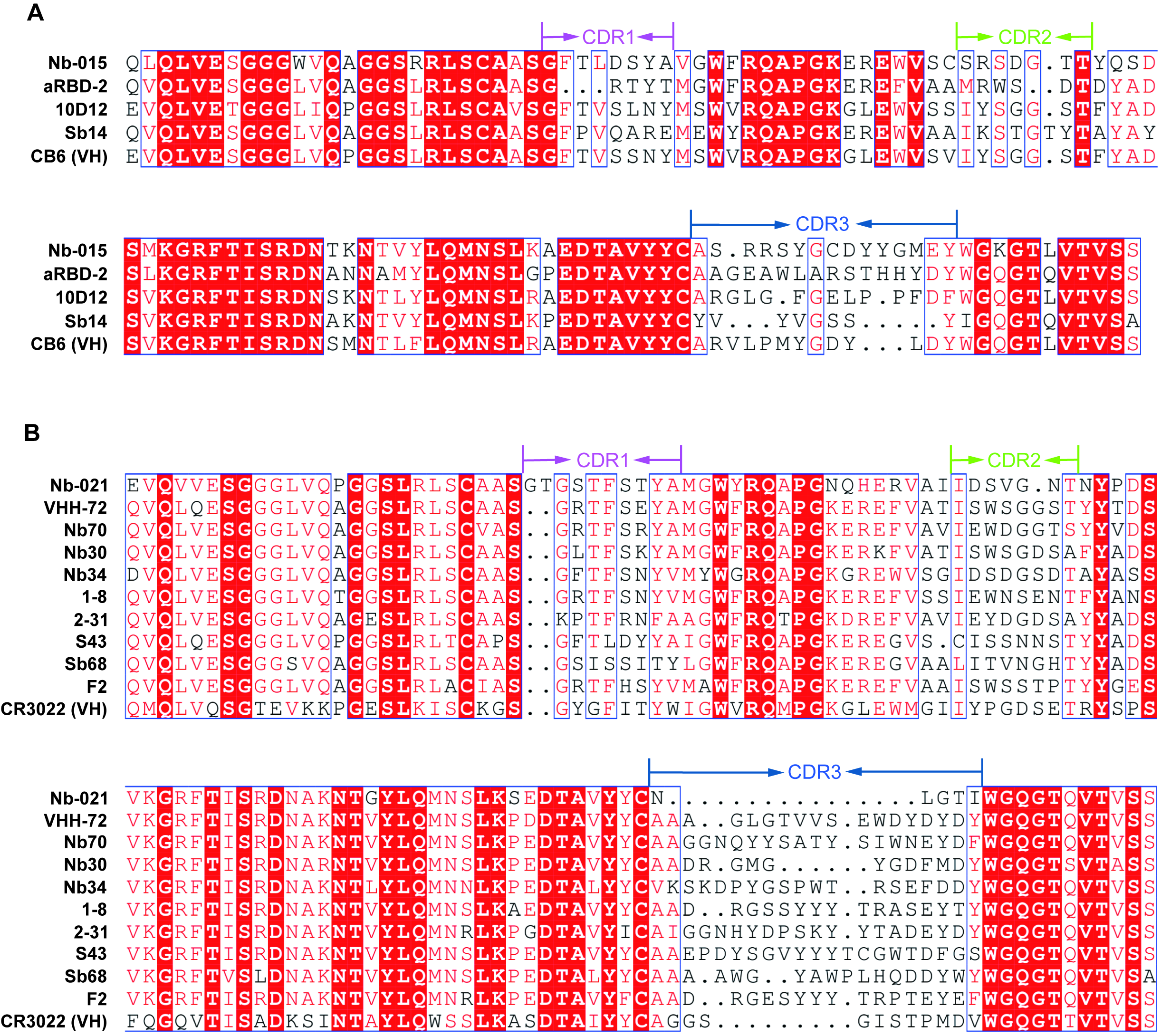

Supplement: S13 Fig — A, Sequence alignment of Nb-015, CB6 antibody (the heavy chain) and three nanobodies (aRBD-2, 10D12 and Sb14) that target similar binding site in S-RBD. The CDR regions are marked. B, Sequence alignment of Nb-021, CR3022 antibody (the heavy chain) and nine nanobodies (VHH-72, Nb70, Nb30, Nb34, 1–8, 2–31, S43, Sb68 and F2) that target similar binding site in S-RBD. The CDR regions are marked. (TIF) [file ppat.1011804.s013.tif]
