# Supplementary material for: Development of a bispecific nanobody conjugate broadly neutralizes diverse SARS-CoV-2 variants and structural basis for its broad neutralization
Source: PLoS Pathog. 2023 Nov 30;19(11):e1011804. doi: 10.1371/journal.ppat.1011804 (PMC10688893; doi:10.1371/journal.ppat.1011804)
Supplement: S1 Table — (DOCX) [file ppat.1011804.s014.docx]

**S1 Table. Data collection and structure refinement statistics.**

|  | SARS-CoV-2 S-RBD  with Nb-015 | SARS-CoV-2 S-RBD  with Nb-021 |
| --- | --- | --- |
| **Data collection** |  |  |
| Space group | *I*4_1_ | *P*2_1_2_1_2_1_ |
| Cell dimensions |  |  |
| *a*, *b*, *c* (Å) | 75.82, 75.82, 119.83 | 78.07, 104.83, 107.05 |
| *α*, *β*, *γ* (°)  Wavelength (Å) | 90, 90, 90  0.97852 | 90, 90, 90  0.97852 |
| Resolution (Å) | 50.00-2.00 (2.07-2.00) | 50.00-2.40 (2.49-2.40) |
| Unique reﬂections | 22,938 (2,299) | 33,895 (3,240) |
| *R*_merge_ | 0.110 (0.664) | 0.173 (0.722) |
| *I*/sig*I* | 46.14 (4.50) | 25.69 (4.20) |
| Completeness (%) | 100.0 (100.0) | 97.9 (95.7) |
| Redundancy | 13.2 (13.4) | 11.9 (11.5) |
|  |  |  |
| **Refinement** |  |  |
| Resolution (Å) | 26.15-2.00 | 40.69-2.40 |
| No. reflections | 22,918 | 33,843 |
| *R*_work_/*R*_free_ | 0.171/0.202 | 0.226/0.246 |
| No. of atoms |  |  |
| Protein | 2,420 | 4,848 |
| Ligand/ion | 0 | 0 |
| Water | 146 | 308 |
| *B*-factors (Å^2^) |  |  |
| Protein | 28.5 | 39.6 |
| Ligand/ion | - | - |
| Water | 39.1 | 41.0 |
| R.m.s. deviations |  |  |
| Bond lengths (Å) | 0.008 | 0.004 |
| Bond angles (°) | 0.905 | 0.734 |
| Ramachandran plot (%)  Favored region  Allowed region  Outlier region | 97.98  2.02  0 | 97.71  2.29  0 |
| **PDB code** | 8H5T | 8H5U |

A single crystal was used to collect the data.

Values in parentheses are for the highest-resolution shell.
