# Supplementary material for: Development of a bispecific nanobody conjugate broadly neutralizes diverse SARS-CoV-2 variants and structural basis for its broad neutralization
Source: PLoS Pathog. 2023 Nov 30;19(11):e1011804. doi: 10.1371/journal.ppat.1011804 (PMC10688893; doi:10.1371/journal.ppat.1011804)
Supplement: S2 Table — (DOCX) [file ppat.1011804.s015.docx]

**S2 Table. Summary of the atomic binding details between Nb-021 and SARS-CoV-2 S-RBD.**

| Nb-021^a^ | | | Contacts with SARS-CoV-2  S-RBD amino acids^b^ | Total contacts^c^ | Proportion^d^ |
| --- | --- | --- | --- | --- | --- |
| Framework region | | Y39 | Y369 (7), F377 (1), P384 (3) | 260 | 88.7% |
|  |  | N45 | C379 (2), Y380 (4), G381 (11),  V382 (5) |  |  |
|  |  | Q46 | K378 (1), C379 (5), Y380 (11) |  |  |
|  |  | H47 | F377 (1), K378 (7), C379 (12),  V382 (5), S383 (14), P384 (7) |  |  |
|  |  | E48 | F377 (2), K378 (9) |  |  |
|  |  | R49 | Y369 (6), N370 (3),  S371 (5), F374 (5), F377 (11) |  |  |
|  |  | I52 | Y369 (2), N370 (7), S371 (6), A372 (4) |  |  |
|  |  | N60 | A372 (5), S373 (2), F374 (10),  S375 (10) |  |  |
|  |  | Y61 | S375 (9) |  |  |
|  |  | P62 | S375 (7) |  |  |
|  |  | Y96 | P384 (4), T385 (3) |  |  |
|  |  | W103 | Y369 (12), P384 (13), T385 (29) |  |  |
| CDRs | CDR1 | A35 | N370 (7) | 33 | 11.3% |
|  | CDR2 | N58 | A372 (3) |  |  |
|  |  | T59 | A372 (5) |  |  |
|  | CDR3 | N98 | Y369 (2), N370 (4) |  |  |
|  |  | G100 | Y369 (2), N370 (5) |  |  |
|  |  | T101 | S366 (2), Y369 (3) |  |  |

^a^ The interface residues in Nb-021 and the structural regions to which these identified amino acids belong are listed (residues providing ≥2 contacts are shown, the distance cutoff is 4.5 Å).

^b^ Numbers in parentheses represent the number of van der Waals contacts between the indicated Nb-021 and SARS-CoV-2 S-RBD residues (the distance cutoff is 4.5 Å).

^c^ Total contacts indicate the sum of the van der Waals contacts for the CDRs or framework region in Nb-021.

^d^ Proportion indicates the percentage of the van der Waals contacts provided by CDRs or framework region in that provided by all the interface residues.
