# Supplementary material for: Development of a bispecific nanobody conjugate broadly neutralizes diverse SARS-CoV-2 variants and structural basis for its broad neutralization
Source: PLoS Pathog. 2023 Nov 30;19(11):e1011804. doi: 10.1371/journal.ppat.1011804 (PMC10688893; doi:10.1371/journal.ppat.1011804)
Supplement: S3 Table — (DOCX) [file ppat.1011804.s016.docx]

S3 Table. **Summary of the IC_50_ values of mono-, bi-, and tetra-valent nanobodies against SARS-CoV-2 pseudoviruses.**

“ND” represents “not determined”.

| SARS-CoV-2 pseudovirus | IC_50_ (nM) | | | | |
| --- | --- | --- | --- | --- | --- |
|  | Nb-015 | Nb-021 | Nb-015-Fc | Nb-021-Fc | Nb-X2-Fc |
| Original strain | 164 | 61.1 | 0.421 | 4.21 | 1.89 |
| Alpha (B.1.1.7) | 95.6 | 33.7 | 0.864 | 4.72 | ND |
| Beta (B.1.351) | 301 | 50.3 | 0.648 | 3.26 | 0.727 |
| Gamma (P.1) | 1259 | 38.3 | 44.3 | 6.08 | 1.44 |
| Delta (B.1.617.2) | 130 | 16.8 | 0.381 | 6.34 | 5.08 |
| Kappa (B.1.617.1) | 79.1 | 31.4 | 0.598 | 3.93 | ND |
| Lambda (C.37) | 66.4 | 19.7 | 0.359 | 3.58 | ND |
| Mu (B.1.621) | 79.0 | 21.9 | 0.250 | 1.84 | ND |
| Omicron (BA.2) | 697 | 1.91e+4 | 3.31 | 95.7 | 2.28 |
| Omicron (BA.4/BA.5) | 135 | 1.78e+4 | 1.17 | 24.3 | 2.14 |
| Omicron (XBB.1.5) | ND | ND | ~3.97e+4 | ~1.59e+5 | 87.2 |
| Omicron (XBB.1.16) | ND | ND | ~6.80e+4 | ~2.44e+5 | 142 |
